# An individualized immune signature of pretreatment biopsies predicts pathological complete response to neoadjuvant chemoradiotherapy and outcomes in patients with esophageal squamous cell carcinoma

**DOI:** 10.1038/s41392-020-00221-8

**Published:** 2020-09-04

**Authors:** Chaoqi Zhang, Guochao Zhang, Nan Sun, Zhen Zhang, Liyan Xue, Zhihui Zhang, Haijun Yang, Yuejun Luo, Xiaoli Zheng, Yonglei Zhang, Yufen Yuan, Ruixue Lei, Zhaoyang Yang, Bo Zheng, Le Wang, Yun Che, Feng Wang, Sihui Wang, Shugeng Gao, Qi Xue, Yi Zhang, Jie He

**Affiliations:** 1 Department of Thoracic Surgery, National Cancer Center/National Clinical Research Center for Cancer/Cancer Hospital, Chinese Academy of Medical Sciences and Peking Union Medical College, Beijing, 100021 China; 2 Biotherapy Center, The First Affiliated Hospital of Zhengzhou University, Zhengzhou, 450052 Henan China; 3 Department of Pathology, National Cancer Center/ National Clinical Research Center for Cancer/Cancer Hospital, Chinese Academy of Medical Sciences and Peking Union Medical College, Beijing, 100021 China; 4 Department of Pathology, Anyang Cancer Hospital, The Fourth Affiliated Hospital of Henan University of Science and Technology, Anyang, 455000 Henan China; 5 Department of Radiotherapy, The Affiliated Cancer Hospital of Zhengzhou University, Zhengzhou, 450008 Henan China; 6 Department of General Surgery, The Affiliated Cancer Hospital of Zhengzhou University, Zhengzhou, 450008 Henan China; 7 Department of Otology, The First Affiliated Hospital of Zhengzhou University, Zhengzhou, 450052 Henan China

**Keywords:** Gastrointestinal cancer, Tumour immunology, Predictive markers, Tumour biomarkers

## Abstract

No clinically available biomarkers can predict pathological complete response (pCR) for esophageal squamous cell carcinomas (ESCCs) with neoadjuvant chemoradiotherapy (nCRT). Considering that antitumor immunity status is an important determinant for nCRT, we performed an integrative analysis of immune-related gene profiles from pretreatment biopsies and constructed the first individualized immune signature for pCR and outcome prediction of ESCCs through a multicenter analysis. During the discovery phase, 14 differentially expressed immune-related genes (DEIGs) with greater than a twofold change between pCRs and less than pCRs (<pCRs) were revealed from 28 pretreatment tumors in a Guangzhou cohort using microarray data. Ten DEIGs were verified by qPCR from 30 cases in a Beijing discovery cohort. Then, a four-gene-based immune signature (*SERPINE1*, *MMP12*, *PLAUR*, and *EPS8*) was built based on the verified DEIGs from 71 cases in a Beijing training cohort, and achieved a high accuracy with an area under the receiver operating characteristic curve (AUC) of 0.970. The signature was further validated in an internal validation cohort and an integrated external cohort (Zhengzhou and Anyang cohorts) with AUCs of 0.890 and 0.859, respectively. Importantly, a multivariate analysis showed that the signature was the only independent predictor for pCR. In addition, patients with high predictive scores showed significantly longer overall and relapse-free survival across multiple centers (*P* < 0.05). This is the first, validated, and clinically applicable individualized immune signature of pCR and outcome prediction for ESCCs with nCRT. Further prospective validation may facilitate the combination of nCRT and immunotherapy.

## Introduction

Esophageal cancer (EC) is the sixth most common cause of cancer-related mortality with 509,000 deaths occurring annually worldwide.^1^ EC contains two major histological types: squamous cell carcinoma and adenocarcinoma.^2^ Although the incidence of esophageal adenocarcinoma in the Western world has risen sixfold over the last 40 years,^3,4^ esophageal squamous cell carcinoma (ESCC) remains prevalent in Asia, especially in China, where it accounts for more than 90% of cases of EC and makes up almost half of the global disease burden.^5^ In patients with resectable disease, the combined modality approach of perioperative neoadjuvant chemoradiotherapy (nCRT) and esophagectomy has become the standard treatment option. This combined approach is associated with a modest superior overall survival (OS) compared with surgery alone for the treatment of locally advanced ESCC.^6–8^ However, the clinical outcomes of ESCCs after nCRT are heterogeneous. In fact, only patients who achieve a pathological complete response (pCR)—defined as a pathological examination which features no tumor cells, regardless of the resected primary tumor site or the lymph nodes of the surgical specimens—have significantly improved survival and are therefore recognized as responders.^9^ The percentage of pCRs in the esophagectomy specimens after nCRT is only about 20–40%,^7,10^ indicating that more than half of the patients were identified as less than pCR (<pCR). The <pCRs are classified as nonresponders to nCRT and considered less likely to benefit from this regimen. Moreover, these nonresponders who received nCRT followed by surgery showed higher morbidity and mortality rates than those who only underwent surgery.^11^ Therefore, the identification of biomarkers available for predicting pCRs represents an urgent need and may help practitioners select the appropriate treatment for patients with ESCCs.

The advent of immunotherapies has greatly changed the treatment landscape across a variety of malignancies, including ECs. This is particularly true for immune checkpoint inhibitors (ICIs) which target the interaction of programmed cell death 1 and programmed cell death-ligand 1 (PD-L1).^12,13^ Although immunotherapy is broadly active and is regarded as a new hope for many cancers, a considerable number of patients were found with de novo or acquired resistance.^14,15^ Given the moderate antitumor efficacy of immunotherapy and the relatively extensive resistance, a combination treatment of immunotherapy and other therapeutic strategies—designed to recruit more immune cells into the tumor—is considered as an effective approach for improving treatment efficiency. Clinical trials of these combination treatments are currently ongoing throughout the world.^16,17^

It is well known that chemoradiotherapy can activate the immune system via various mechanisms—including initiating immunogenic cell death, promoting the production and release of inflammatory factors into the tumor microenvironment (TME), aggrandizing the expression and presentation of tumor antigens, and by facilitating the infiltration of multiple immune cells, which may help to overcome the immunosuppressive effects of the TME.^18,19^ Several studies have confirmed that nCRT is also closely related to immunogenomic changes in the tumor and the TME, especially in ESCCs.^20,21^ Moreover, preliminary results of the latest phase II clinical trials about combining ICIs with nCRT in ESCCs showed promising efficacy with acceptable toxicity.^22^ Recently, studies revealed that the expression of immune checkpoint molecules like PD-L1 and indoleamine 2,3-dioxygenase 1 from pretreatment endoscopic cancer biopsies were biomarkers that could predict pathologic response after nCRT in ESCCs.^23,24^ Thus, discrepancies in immune molecular profiles, observed in different cases, may potentially comprise powerful models for nCRT prediction. However, little is known about the landscape of immune molecules in the pretreatment specimens between pCRs and <pCRs. In addition, more information is needed on the prognostic value of these profiles in patients with ESCCs who undergo nCRT.

The primary aim of this study was to identify and validate an immune signature in a large number of pretreatment endoscopic cancer biopsies to predict pCR and outcomes of ESCCs treated with nCRT. This was the first and largest retrospective analysis of patients with ESCCs, and involved multiple centers in China. Data contributed by each center were used to build an immune-specific signature for nCRT prediction. The study cohort consisted of 252 cases from four hospitals in three different districts in China with a high incidence of ESCC (Guangdong, Hebei, and Henan).^25,26^ Eventually, an immune-related signature based on four genes (*SERPINE1*, *MMP12*, *PLAUR*, and *EPS8*) with real-time quantitative polymerase chain reaction (qPCR) value was constructed and well-validated in multiple institutions. Notably, our immune-related signature was the first mRNA model to show strong prognostic accuracy for ESCCs treated with nCRT. A better understanding of the immune-related panorama of the pretreatment samples between pCRs and <pCRs will provide a foundation for the further combination of nCRT and immunotherapy while optimizing treatment individualization and prognostic management of patients with ESCCs.

## Results

### Patients’ characteristics

The detailed clinicopathological characteristics of enrolled patients in the discovery cohort, training cohort, internal validation cohort, and integrated external validation cohort are summarized in Table 1. Totally, 252 ESCC cases with nCRT before surgery from multicenter were collected. Postoperative pathological examination exhibited that pCR was confirmed in 34.1% of the total multicenter samples (86 of 252), including 39.3% of the Guangzhou cohort (11 of 28), 36.7% of the Beijing discovery cohort (11 of 30), 32.4% of the Beijing training cohort (23 of 71), 33.8% of the Beijing validation cohort (24 of 31), and 32.7% of the integrated external validation cohort (17 of 52). Besides, the OS data of the total and Beijing sub-cohorts, as well as the integrated external validation cohort, were collected to evaluate the prognosis between pCRs and <pCRs. Kaplan–Meier analyses revealed that pCRs showed a tendency toward better OS in the different cohorts (Supplementary Data Fig. S1). In addition, we also collected relapse-free survival (RFS) data from the Beijing cohort. Similarly, worse RFS in the <pCR groups were shown across all different cohorts (Supplementary Data Fig. 2).

**Table 1:** Clinical characteristics of enrolled patients from the multicenter cohorts

	Discovery cohort		Training cohort	Internal validation cohort	External validation cohort
	Guangzhou cohort	Beijing discovery cohort	Beijing training cohort	Beijing validation cohort	Integrated external validation cohort
	(*N* = 28)	(*N* = 30)	(*N* = 71)	(*N* = 71)	(*N* = 52)
Age					
≥60	8	17	32	38	40
<60	20	13	39	33	12
Sex					
Male	25	28	64	63	37
Female	3	2	7	8	15
Tumor location					
Upper	4	3	19	18	14
Middle	18	19	40	38	31
Lower	6	8	12	15	7
Tumor differentiation					
Well	7	3	6	4	15
Moderate	16	20	37	36	22
Poor	5	7	28	31	15
Clinical T stage					
T2	8	2	3	6	13
T3	20	11	45	41	32
T4	0	16	23	24	7
Clinical N stage					
N0	0	3	9	14	25
N1, N2, N3	28	27	62	57	27
Clinical M stage					
M0	28	30	71	71	52
M1	0	0	0	0	0
Clinical TNM stage					
II	8	5	8	16	26
III	20	25	63	55	26
nCRT response					
pCR	11	11	23	24	17
<pCR	17	19	48	47	35

*nCRT* neoadjuvant chemoradiotherapy, *pCR* pathological complete response, *<pCR* less than pCR

### Identification and validation of differentially expressed immune-related genes (DEIGs) from pretreatment biopsies between pCRs and <pCRs

To clarify the immune molecular profiles between pCRs and <pCRs in the pretreatment biopsies of ESCCs, we first downloaded 3193 immune-related genes from AmiGO 2 and obtained 2695 matched genes (Supplementary Data Table S3) in the Guangzhou cohort. After log2 transformation, the average expression level of these 2695 immune-related genes in 28 pretreatment samples was 8.473. To better apply our model to clinical practice, we focused on the mRNAs with high expression values and filtered out 1313 mRNAs with mean values lower than 8.473. Eventually, 1382 mRNAs with high expression levels were used for further analysis. Then, we identified 14 DEIGs between the pCRs and <pCRs with the screening strategy of fold change >2 and *P* < 0.05, among which twelve mRNAs (*MMP1*, *INHBA*, *SERPINE1*, *KLK5*, *DSG1*, *MMP12*, *MMP9*, *FST*, *LGALS1*, *AIM2*, *PLAUR*, and *CTSV*) were upregulated and two mRNAs (*PTN* and *EPS8*) were downregulated in pCRs (Supplementary Data Fig. 3 and Table S4). Then, these 14 DEIGs were verified in 30 formalin-fixed paraffin-embedded (FFPE) samples, including 11 pCRs and 19 <pCRs, from the Beijing discovery cohort by qPCR. Our results showed that ten genes were significantly differentially expressed between pCRs and <pCRs (*P* < 0.05, Supplementary Data Fig. 4).

### Immune-related predictive signature construction

**Fig. 1:**
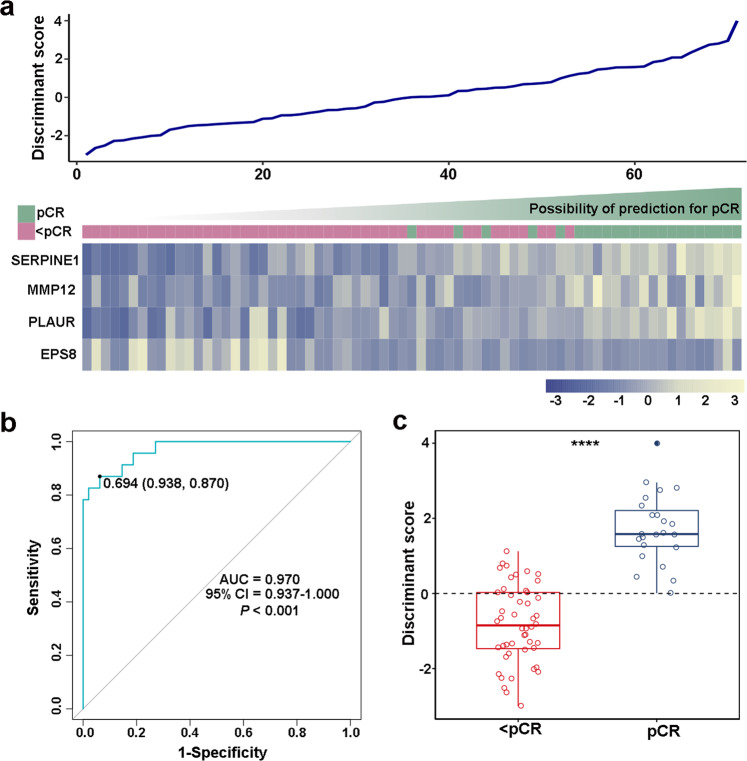
Construction of an individualized immune signature for pCR prediction in patients with ESCCs treated with nCRT. **a** A heatmap of the identified four-gene-based immune signature and the corresponding discriminant score. **b** Receiver operating characteristic curve (ROC) for the performance of the immune signature in the training cohort. **c** The distributions of the discriminant scores between pCRs and <pCRs in the training cohort. **** represents *P* < 0.0001

To build the immune-related signature for prediction of pCRs, we detected the expression profiles of these ten genes in 71 samples from the Beijing training cohort by qPCR. To shrink the number of variables and build a classifying model, Fisher’s linear discriminant analysis (FLDA) with stepwise variant-selection was used based on the log2-transformed qPCR values of the ten genes in the Beijing training cohort to construct the model. Finally, a classifier was established with the equation *Y* = −2.794 + 0.606 × *SERPINE1* + 0.614 × *MMP12* + 0.682 × *PLAUR* − 1.751 × *EPS8* (eigenvalue 1.458, canonical correlation 0.77, *P* < 0.001). The relationship between the expression landscape of these four selected immune-related genes in our signature, and the discriminant score based on the equation are shown in Fig. 1a. With a cut value of 0.694, we found that 20 of 23 were successfully classified as pCRs with a sensitivity of 87.0%. Further, 45 of 48 were correctly classified as <pCRs with a specificity of 93.8%. The overall accuracy of our signature was 91.5% (65 of 71) with an area under the receiver operating characteristic (ROC) curve (AUC) of 0.970 [*P* < 0.001, 95% confidence interval (CI) 0.937–1.000] (Fig. 1b, c). To compare the predictive accuracy of the model with a single variable, we evaluated the predictive power of *SERPINE1*, *MMP12*, *PLAUR*, and *EPS8* in the Beijing training cohort. As excepted, the predictive effect of the immune-related signature was better than any of the signal markers, with AUCs of 0.892, 0.741, 0.835, and 0.709, respectively (Supplementary Data Fig. 5). To preliminarily assess the predictive ability of the immune-related signature, we tested our model in the Guangzhou cohort and Beijing discovery cohort (Supplementary Data Fig. 6). In the Guangzhou cohort, the model successfully identified 24 of 28 samples with an overall accuracy of 85.7% and an AUC of 0.866 (*P* = 0.001, 95% CI 0.727–1.000). Similarly, in the Beijing discovery cohort, our signature demonstrated an overall accuracy of 90.0% and AUC of 0.928 (*P* < 0.001, 95% CI 0.837–1.000). These results initially confirmed that the novel immune-related signature is reliable.

### Validation of the immune-related predictive signature in the internal cohort

**Fig. 2:**
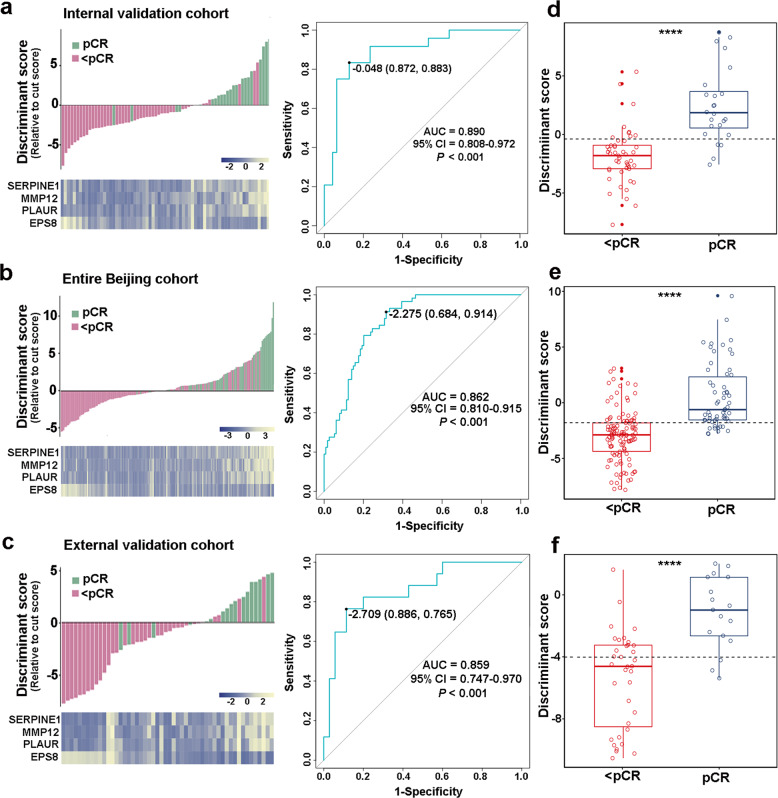
Evaluation of the immune signature in the internal validation cohort, the entire Beijing cohort, and the external validation cohort. A heatmap of the identified four-gene-based immune signature with the corresponding discriminant scores (left panel), and receiver operating characteristic curves (ROC) for the performance of the immune signature (right panel) in the internal validation cohort (**a**), entire Beijing cohort (**b**), and external validation cohort (**c**). Distributions of the discriminant scores between pCRs and <pCRs in the internal validation cohort (**d**), entire Beijing cohort (**e**), and external validation cohort (**f**). **** represents *P* < 0.0001

In the validation phase, we evaluated the performance of quadratic discriminant in 71 cases from the internal Beijing validation cohort, which contained 47 pCRs and 24 <pCRs. We found that the model still worked robustly with a sensitivity of 83.3% and specificity of 87.2%. As shown in Fig. 2a, the overall accuracy of this signature in 71 cases was 86.0%, with an AUC of 0.890 (*P* < 0.001, 95% CI 0.808–0.972). To further validate the signature in the entire internal Beijing cohort, we combined the Beijing discovery, training, and validation cohorts. Our results, as shown in Fig. 2b, showed that the classifier performed well in the 172 cases from the entire Beijing cohort with an overall accuracy of 76.2% and AUC of 0.862 (*P* < 0.001, 95% CI 0.810–0.915). The distributions of discriminant scores between pCRs and <pCRs in the Beijing validation cohort and the entire Beijing cohort are shown in Fig. 2d, e (*P* < 0.05).

### Validation of the immune-related predictive signature in the integrated external cohort

To determine whether the immune-related signature could be reproduced in the Chinese population, we integrated two independent institutions—the Zhengzhou cohort and Anyang cohort, from an ESCC high-incidence district (Henan, China)^25^—as the integrated external validation cohort. Our results revealed a sensitivity of 76.5% and we successfully identified 13 of 17 pCRs. In addition, we successfully identified 31 of 35 <pCRs with a specificity of 88.6%. Totally, as shown in Fig. 2c, the overall accuracy was 84.6% (44 of 52) and the AUC was 0.859 (*P* < 0.001, 95% CI 0.747–0.970). As shown in Fig. 2f, a significant difference was found in the discriminant scores between pCRs and <pCRs in the external cohort (*P* < 0.0001). We also tested the signature in the Zhengzhou cohort and Anyang cohort, respectively. As expected, the signature was still robust in the independent cohorts, with AUCs of 0.833 and 0.925, respectively (Supplementary Data Fig. 7). Collectively, these data suggested that the immune-related signature is stable to predict pCRs of ESCCs treated with nCRT from different institutions across China.

### Factors associated with pCR after nCRT

To determine the factors that contributed to pCR following nCRT, we collected age, sex, tumor location, tumor differentiation, pretreatment clinical TNM stage, chemotherapy regimen, and the immune-related signature. Univariate logistic regression analysis was applied, and we found that the immune-related signature score was the only factor that significantly correlated with pCR across the Beijing training cohort, Beijing validation cohort, entire Beijing cohort, and integrated validation cohort (*P* < 0.05, Table 2). Moreover, multivariate logistic regression analysis revealed that the immune-related signature score was the only independent factor, after adjustment for other parameters, that was significantly associated with pCR in the multicenter cohorts (*P* < 0.05, Table 2).

**Table 2:** Univariate and multivariate analyses of various predictive factors for pCR in different cohorts

		Univariable analysis	Multivariable analysis		
		*P* value^a^	*P* value^b^	OR	95% CI
Beijing training cohort					
Age	≥60/<60	0.406			
Sex	Male/female	0.156			
Tumor location	Upper, middle/lower	0.820			
Tumor differentiation	Moderately, poorly/well differentiated	0.345			
Clinical TNM stage	II/III	0.820			
Chemotherapy regimen^c^	1/2, 3	0.629			
Discriminant score	High/low	<0.001	<0.001	171.873	20.259–1458.122
Beijing validation cohort					
Age	≥60/<60	0.562			
Sex	Male/female	0.579			
Tumor location	Upper, middle/lower	0.240			
Tumor differentiation	Moderately, poorly/well differentiated	0.489			
Clinical TNM stage	II/III	0.125			
Chemotherapy regimen^c^	1/2, 3	0.309			
Discriminant score	High /low	<0.001	<0.001	153.824	12.166–1944.861
Entire Beijing cohort					
Age	≥60/<60	0.592			
Sex	Male/female	0.226			
Tumor location	Upper, middle/lower	0.554			
Tumor differentiation	Moderately, poorly/well differentiated	0.120			
Clinical TNM stage	II/III	0.317			
Chemotherapy regimen^c^	1/2, 3	0.398			
Discriminant score	High /low	<0.001	<0.001	26.169	9.075–75.458
External validation cohort					
Age	≥60/<60	0.957			
Sex	Male/female	0.222			
Tumor location	Upper, middle/lower	0.153			
Tumor differentiation	Moderately, poorly/well differentiated	0.010	0.145	0.235	0.034–1.645
Clinical TNM stage	II/III	0.043	0.068	0.111	0.010–1.177
Chemotherapy regimen^c^	1/2, 3	0.629			
Discriminant score	High/low	<0.001	0.001	56.133	4.804–655.843

*pCR* pathological complete response, *OR* odds ratio, *CI* confidence interval

^a^*χ*^2^ or Fisher exact tests

^b^Logistic regression analysis with a forward stepwise procedure and likelihood ratio test

^c^1, platinum/paclitaxel; 2, platinum/fluorouracil; 3, platinum/others

### Prognostic value of the immune-related signature

**Fig. 3:**
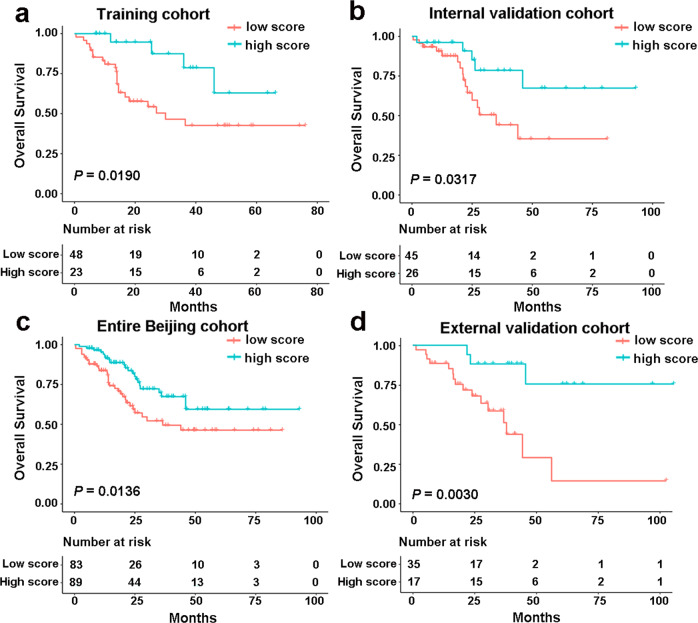
The performance of the immune signature in predicting outcome in ESCC with nCRT. Kaplan–Meier survival curves for OS based on the discriminant scores in training cohort (**a**), internal validation cohort (**b**), entire Beijing cohort (**c**), and the external validation cohort (**d**)

Patients who achieved pCR after nCRT had a significant survival advantage compared to patients who were classified as <pCR.^9^ We can therefore assume that our immune-related signature can be used for survival prediction in patients with ESCCs treated with nCRT. To verify our assumption, we first evaluated the relationship between the immune-related signature score and OS in the Beijing training cohort. Kaplan–Meier survival analyses revealed that patients in the high discriminant score group had significantly longer survival (Fig. 3a, *P* = 0.0190, HR 0.3035, 95% CI 0.1372–0.6716). To confirm what we found in the training cohort, we explored the model in the Beijing validation cohort. As expected, with the cut point of −0.048, patients in the low score group had a significantly higher mortality risk than patients in the high score group (Fig. 3b, *P* = 0.0317, HR 0.3545, 95% CI 0.1504–0.8353). Furthermore, the same score formula and OS data were used in the entire Beijing cohort to further validate the signature’s prognostic ability. Similarly, the OS time of the high score group was significantly longer than the low score group (Fig. 3c, *P* = 0.0136, HR 0.5128, 95% CI 0.2991–0.8793). Finally, a survival analysis in the external validation cohort also confirmed that, in patients with a high discriminate score, the OS was significantly longer than that in patients with low discriminate scores (Fig. 3d, *P* = 0.0030, HR 0.1994, 95% CI 0.0811–0.4903).We also analyzed the relationship between the classifier and RFS. Consistent with the OS results, results from Kaplan–Meier analysis revealed that patients with a higher discriminate score had a significantly better RFS than those with a lower discriminate score in the different cohorts (Supplementary Data Fig. 8, *P* < 0.05).

## Discussion

ESCC is one of the most aggressive tumor types and is associated with a high mortality rate in China.^2^ Patients with ESCCs usually present with advanced stage at the time of diagnosis. In these patients, nCRT followed by surgery has shown superior clinical outcomes.^7^ However, the treatment effects of this therapeutic regimen are heterogeneous, and current methods are insufficient to predict nCRT responders. NCRT can generate an immune response with increased presentation of antigens and immune components. These components include immune checkpoints and tumor-infiltrating lymphocytes—newly identified biomarkers for pCRs.^24,27,28^ Therefore, we assumed that the immune-related signature may be able to predict response to nCRT in patients with ESCCs. Herein, we conducted the largest multicenter retrospective analysis of patients with ESCC to date and built an immune-specific signature for nCRT prediction, which contained 244 cases from four hospitals in three different ESCC high-incidence districts in China.

To the best of our knowledge, this is the first and most comprehensive study to date demonstrating the prognostic accuracy of an immune signature in patients with ESCC undergoing nCRT. Examination of immune-specific signatures from pretreatment endoscopic samples taken from pCRs and <pCRs may provide additional novel perceptions into the synergistic effects of immunotherapy and nCRT, and reveal potentially predictive immune biomarkers.

We analyzed all the immune-related genes from pretreatment cancer biopsies and selected those that were differentially expressed in pCRs and <pCRs. Then we gathered 14 immune-related genes at high expression levels, with greater than a twofold change. Finally, ten immune-related genes were well-validated by qPCR in an independent discovery cohort. Using these verified genes, we identified a four-genes-based immune signature by FLDA with stepwise variant-selection from 71 cases in the Beijing training cohort. This signature provided an overall accuracy of 91.5% and an AUC of 0.970. Then the diagnostic accuracy of immune-related signature was well-validated with high accuracy in an internal validation cohort, with the overall accuracy of 86.0% and an AUC of 0.890. In addition, during the validation of the entire Beijing cohort, the immune-related signature also showed a robust prediction accuracy of 76.2% and an AUC of 0.862.

To verify the universality of our signature for the Chinese population, we enrolled two external cohorts as the integrated external validation cohort. The rates of ESCCs vary as much as tenfold among the districts within China. In addition, there are dramatic differences over short geographic distances.^2^ Lin county (Linxian) is the most studied region of China and located in the North Central Taihang Mountain range along the northern border of Henan Province. Here, ESCC is a leading cause of death, with incidence rates exceeding 125/100,000 per year.^2,26^ Therefore, we selected two cohorts consisting of patients from Linxian and other regions in the Henan Province—the Zhengzhou cohort and Anyang cohort—as the most representative integrated external cohort. As expected, our signature was well-validated in both the integrated external cohort and the two separate external cohorts. These findings indicated that our novel immune signature could predict pCR of ESCC with nRCT in multicenter Chinese cohorts. We also demonstrated that the signature was a novel independent risk factor for patients with ESCC undergoing nCRT in multiple institutions. These findings suggested that antitumor immunity was involved in the response to nCRT, regardless of the patient’s clinicopathological factors or the institution’s chemotherapy regimen.

As far as the ultimate objective that prediction model is concern, the prediction for patients’ survival is just the ideal condition. To achieve this objective, we collected the OS and RFS data of cases from multiple centers and explored the prognostic significance of the immune signature for ESCCs with nCRT. As expected, the four-gene signature was able to divide patients into high- and low-discriminant score groups. These groups demonstrated significantly different rates of survival in different independent centers across China. This suggests that our immune signature has great potential in clinical practice for early management of prognosis in patients with ESCC being treated with nCRT.

In this study, four immune-related genes—*SERPINE1*, *MMP12*, *PLAUR*, and *EPS8*—were recruited as part of the novel immune-related signature to distinguish pCRs from <pCRs. *SERPINE1*, an endothelial plasminogen activator inhibitor, also known as *PAI-1*, was reported widely expressed in various cancers and closely related to patients’ outcomes.^29,30^ Interestingly, Ostheimer et al. pointed out that low *PAI-1* levels were associated with a significantly reduced OS and PFS in patients with lung cancer undergoing radiotherapy.^31^ This stands in accordance with our results. We found that high expression *PAI-1* was enriched in pCRs and associated with improved survival. In addition, *PAI-1* could promote the recruitment and polarization of macrophages in TME.^32^ The high infiltration of macrophages in pretreatment samples was found to be associated with a poor response to neoadjuvant chemotherapy.^33^ Therefore, the specific role of *PAI-1* in the process of nCRT of ESCC requires further exploration. *MMP12* is a member of the matrix metalloproteinase (MMP) family, whose members are well known for their essential roles in tumor invasiveness and multidrug resistance.^34^ Interestingly, a recently study pointed out that knocking out *MMP12* caused the accumulation of macrophages in the TME,^35^ indicating that knocking out *MMP12* may enhance chemoradiotherapy resistance in a macrophage-mediated way. This is in line with our finding that *MMP12* was highly expressed in pCRs with a better response to nCRT. *PLAUR* is also known as *UPAR*, which reportedly plays an important role through the activation of latent growth factors, degradation of the extracellular matrix, and involvement in drug resistance.^36^ Besides, *UPAR* promoted tumor-permissive conditioning of macrophages and mediates T-cell suppression.^37,38^ This means that high *UPAR* may be related to chemoradiotherapy resistance. However, *UPAR* was found predominantly expressed in pretreatment samples from pCRs in our system. Hence, more research is needed to determine the specific role of *UPAR* in the process of nCRT for ESCCs. *EPS8*, a cytoplasmic protein that acts as a substrate of receptor and non-receptor tyrosine kinases, has been identified as an oncogene and plays a crucial role in several tumor types.^39^ What’s more, *EPS8* knockdown was related to increased chemosensitivity in several different cancer cell lines.^39,40^ Moreover, Wang et al. reported that overexpression of *EPS8* could upregulate the expression level of the chemokine ligand CXCL5.^41^ Further, CXCL5 is well known for its ability to recruit neutrophils.^42^ The association of the intra-tumoral infiltration of neutrophils with a poor response to chemoradiotherapy has been revealed by several investigators.^43,44^ These findings were incompatible with our results as we found that high expression of *EPS8* was found in <pCRs and associated with resistance to nCRT. Although the functions of these genes in tumor progression and drug resistance have been reported, the combination and function of these genes in nCRT sensitive and resistant groups of patients with ESCCs remain unknown. This relationship requires further investigation.

Before our study, several studies established molecular signatures from pretreatment endoscopic samples to predict pathological response in patients with EC.^45–50^ However, few studies have paid attention to squamous histology.^49,50^ Others were focused on adenocarcinoma-dominated mixed histories. In fact, these limited predictive signatures are insufficient for application in clinical practice owing to sample size limitations, lack of prognostic data, and lack of external validation. Compared with previous squamous cell carcinoma-specific studies, our research has several novelties and advantages. First, with AUCs of 0.970 and 0.890 in the training cohort and internal validation cohort, respectively, the predictive powers of our immune signature were better and more stable than previously reported ESCC nCRT response prediction models. These models demonstrated AUCs of 0.82–0.87 in the internal validation cohort.^49,50^ Second, our formula was the first mRNA-based signature that was well-validated in different independent cohorts with a total sample size that far outnumbered any previous studies. This provides much more creditability and reliability for clinical practice. Finally, survival prediction was fulfilled in our mRNA-based prediction model, suggesting that our signature is more suitable for long-term treatment effect evaluation.

Several limitations of our study should also be acknowledged. First, our research was a retrospective cohort study based on FFPE samples from different institutions. Future studies should examine fresh samples prospectively. Second, because of the inevitable RNA degradation in FFPE samples, it was difficult to obtain satisfactory samples of significant size at endoscopy. Therefore, the number of cases in our study was not as large as we expected, especially in the external validation cohorts. Third, the predictive ability of our four-gene-based immune signature might not be stable for the immune TME, which has high spatial heterogeneity. Hence, more cases from different centers are needed to reevaluate our predictive model.

In conclusion, this study introduces a novel four-gene-based immune signature from endoscopic cancer biopsies by qPCR data. This signature could predict pCR and outcomes for patients with ESCC treated with nCRT, and was feasible and reproducible in patients served by multiple centers in China. More importantly, the well-validated survival prediction ability of our novel signature may help optimize early prognosis management in these patients. Prospective clinical trial-based validation of the signature will further facilitate the implementation of patient-specific combined immunotherapy and nCRT.

## Materials and methods

### Study design and participants

This study was performed according to the Declaration of Helsinki and approved by the Ethics Committee of the Cancer Hospital of the Chinese Academy of Medical Sciences. The requirement for informed consent was waived due to the retrospective nature of this study, and all data were anonymously analyzed.

We sought to explore the landscape of immune molecules in pretreatment specimens taken from pCRs and <pCRs. We then built an immune-related signature to predict pCRs among patients with ESCCs who underwent nCRT. Hence, we only enrolled patients with available FFPE biopsy specimens before nCRT. Our research efforts focused on China, and we obtained data from four hospitals in three different high-incidence districts. In total, we examined 252 cases. These included cases obtained from Sun Yat-sen University Cancer Center in Guangzhou (Guangzhou Cohort), which included 28 fresh pretreatment biopsies from patients largely residing in the Guangdong Province (public data, GSE45670).^49^ The Beijing Cohort was drawn from the National Cancer Center (NCC), Cancer Hospital of the Chinese Academy of Medical Sciences in Beijing and consisted of 172 FFPE blocks of pretreatment biopsies (including 30 cases in the Beijing discovery cohort, 71 cases in the Beijing training cohort, and 71 cases in the Beijing internal validation cohort). These patients largely resided within the Hebei Province. The Zhengzhou Cohort was drawn from the Affiliated Cancer Hospital of Zhengzhou University and contained 29 FFPE blocks of pretreatment biopsies obtained from patients largely residing within the Henan Province. Finally, the Anyang Cohort was drawn from the Anyang Cancer Hospital and consisted of 23 FFPE blocks of pretreatment biopsies from patients largely residing within Linxian, in the Henan Province.

**Fig. 4:**
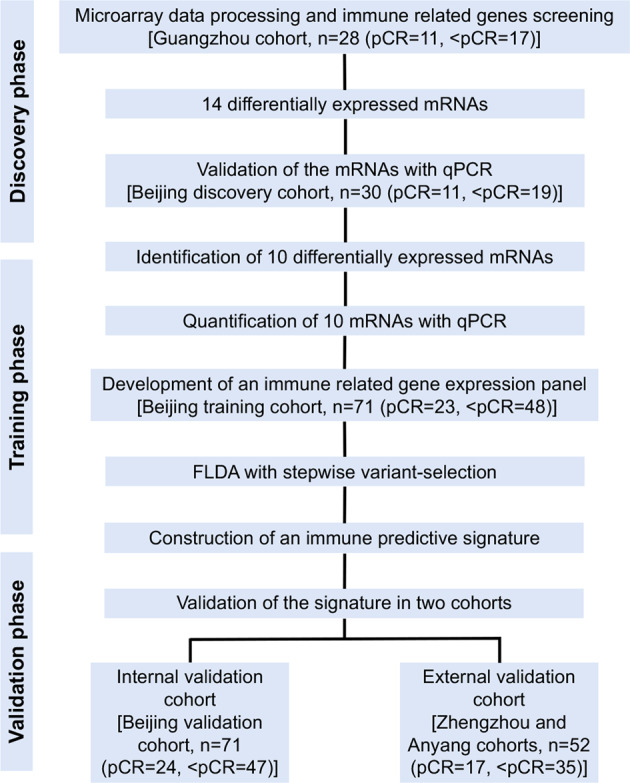
Study flow. The study was performed in multiple institutions, including Guangdong (Sun Yat-sen University Cancer Center), Beijing (National Cancer Center), Zhengzhou (the Affiliated Cancer Hospital of Zhengzhou University), and Anyang (the Anyang Cancer Hospital). pCR pathological complete response, <pCR less than pCR, qPCR real-time quantitative polymerase chain reaction, FLDA Fisher’s linear discriminant analysis

Construction of the immune-related signature took place across three distinct phases. Please refer to the study design, depicted in Fig. 4. In the discovery phase, we screened out DEIGs between pCRs and <pCRs out from microarrays of 28 pretreatment biopsies in the Guangzhou cohort and then carried out validation by qPCR in the Beijing discovery cohort. In the training phase, the qPCR data of validated DEIGs, obtained from 71 cases in the Beijing training cohort, were used to build the signature using FLDA. In the validation phase, the signature was validated in the internal validation cohort and the integrated external validation cohort. Finally, the prognostic value of the signature was also investigated in internal and external cohorts.

### Patients and tissue specimens

Totally, we gathered 252 ESCC cases with available pretreatment biopsies taken before nCRT from four hospitals. The Guangzhou Cohort consisted of a public dataset (GSE45670) with 11 pCRs and 17 <pCRs who underwent treatment from September 2007 to March 2012. The Beijing cohort consisted of 172 cases who underwent treatment from March 2007 to August 2018. The Beijing cohort consisted of three sub-cohorts, including the Beijing discovery cohort with 11 pCRs and 19 <pCRs, the Beijing training cohort with 23 pCRs and 48 <pCRs, and the Beijing validation cohort with 24 pCRs and 47 <pCRs. The Zhengzhou cohort consisted of 9 pCRs and 20 <pCRs who underwent treatment from January 2008 to June 2017. Finally, the Anyang cohort consisted of 8 pCRs and 15 <pCRs who underwent treatment from February 2014 to April 2018. Preoperative nCRT involved external-beam radiotherapy radiation, which typically consisted of overall doses of ~43 Gy (36–50.4 Gy in 18–22 fractions) and concurrent platinum-based chemotherapy. The details of the chemotherapy regimens are included in Supplementary Data Table S1. Esophagectomy was performed to excise the primary tumor and regional nodes ~4–8 weeks after nCRT for patients who were candidates for surgery. We defined the day of surgery to the day of recurrence, metastasis, or last follow-up as the RFS and the day of surgery to the day of death or last follow as the OS. The patients’ characteristics in the multiple instructions were shown in Table 1.

All the clinical pathologic confirmation of the ESCCs from FFPE samples were reevaluated based on the 7th TNM staging system of the American Joint Committee on Cancer. The pathological sections, including the pretreatment and posttreatment samples, were routinely hematoxylin and eosin-stained and independently microscopically assessed by two pathologists. We defined pCR as complete disappearance of tumor cells in the primary tumor site and lymph nodes and <pCR if residual cancer cells were observed. The details of the postoperative pathological responses are also shown in Table 1.

### Publicly available mRNA data and immune gene sets

For discovery cohort, we downloaded 28 cases from the Guangzhou cohort with Gene Expression Omnibus under accession numbers GSE45670 (https://www.ncbi.nlm.nih.gov/geo/query/acc.cgi?acc=GSE45670). The mRNA expression value of GSE45670 was first log2-transformed and quantile-normalized. Genes detected with more than one probe were calculated by mean expression. All the immune-related genes used in this study were gathered from the AmiGO 2 Web portal (http://amigo.geneontology.org/amigo/landing) from searching genes related to immune-related GO terms.

### RNA isolation and qPCR

Only the pretreatment biopsies with at least 80% of tumor cells were enrolled, and 40 μm sections were cut from pretreatment FFPE samples for RNA isolation. RNA was extracted using the Ambion RecoverAll Total Nucleic Acid Isolation Kit for FFPE (ThermoFisher, Waltham, MA, USA) according to the manufacturer’s instructions. RNA quality and quantity were measured by NanoDrop 2000C spectrophotometer (Thermo Scientific, Waltham, MA, USA). Then, 200 ng RNA was used for reverse transcription for 20 μL of reaction, using the FastKing Reverse Transcription Kit (Tiangen Biotech, Beijing, China). Finally, a total of 1 μL cDNA was used for a 10 μL PCR reaction with SYBR in the 7900HT Fast Real-Time PCR System (Applied Biosystems, Carlsbad, IN, USA). The analysis of relative immune-related genes expression was calculated using the 2^−ΔΔCt^ method. Details of the commercially available mRNA primers used for qPCR are included in Supplementary Data Table S2.

### Discrimination analysis

The qPCR expression data of DEIGs in 71 pretreatment samples from the Beijing training cohort were log2-transformed to establish the pCR prediction signature. FLDA, a well-established pattern classification method originally introduced by Fisher,^49^ was then used to construct the model. Using a stepwise approach, the most powerful subset of predicting variables can be defined. Hence, we applied a FLDA with stepwise variant-selection to assess the underlying discrimination ability of DEIGs for pCR in the Beijing training cohort using the SPSS 25.0 software package (SPSS, Chicago, IL). The prediction accuracy of our immune-related signature was calculated by ROC curve analysis.

### Statistical analysis

All the statistical analyses and figures in this study were realized using software R, version 3.5.1 (https://www.r-project.org), and SPSS 25.0 (SPSS, Chicago, IL). The DEIGs were calculated using a moderated *t*-test implemented using the Limma package. The correlations between the clinicopathological parameters or the immune-related signature designated subgroups and pathological responses across multiple centers were analyzed using the *χ*2 or the Fisher exact tests. Logistic regression analysis with a forward stepwise procedure and a likelihood ratio test was conducted to identify independent factors that significantly affected the pathological responses in different cohorts. Other statistical computations and the figures—volcano plot, heatmap, boxplots, ROC curves, and survival curves—were created using several packages (ggplot2, ggrepel, ggthemes, pheatmap, pROC, and survival) in the statistical software environment R. For all statistical methods, a significant difference was declared if the *P* value was < 0.05.

## Supplementary information

Supplementary Materials

## Data Availability

The gene expression data in this study can be found online at the Gene Expression Omnibus under accession numbers GSE45670 (https://www.ncbi.nlm.nih.gov/geo/query/acc.cgi?acc=GSE45670). The other data used and/or analyzed during the current study are available from the corresponding author on reasonable request.
