# Supplementary material for: An individualized immune signature of pretreatment biopsies predicts pathological complete response to neoadjuvant chemoradiotherapy and outcomes in patients with esophageal squamous cell carcinoma
Source: Signal Transduct Target Ther. 2020 Sep 4;5:182. doi: 10.1038/s41392-020-00221-8 (PMC7471268; doi:10.1038/s41392-020-00221-8)
Supplement: Supplementary file 1 — Supplementary Materials [file 41392_2020_221_MOESM1_ESM.docx]

Supplementary Materials for

**An individualized immune signature of pretreatment biopsies predicts pathological complete response to neoadjuvant chemoradiotherapy and outcomes in patients with esophageal squamous cell carcinoma**

Chaoqi Zhang^#1^, Guochao Zhang^#1^, Nan Sun^#1*^, Zhen Zhang^#2^, Liyan Xue^3^, Zhihui Zhang^2^, Haijun Yang^4^, Yuejun Luo^1^, Xiaoli Zheng^5^, Yonglei Zhang^6^, Yufen Yuan^4^, Ruixue Lei^4^, Zhaoyang Yang^3^, Bo Zheng^3^, Le Wang^7^, Yun Che^1^, Feng Wang^1^, Sihui Wang^1^, Shugeng Gao^1^, Qi Xue^1^, Yi Zhang^2^* and Jie He^1*^

#These authors contributed equally: Chaoqi Zhang, Guochao Zhang, Nan Sun, Zhen Zhang

Correspondence to: Nan Sun (sunnan@vip.126.com) or Yi Zhang (yizhang@zzu.edu.cn) or Jie He (prof.jiehe@gmail.com)


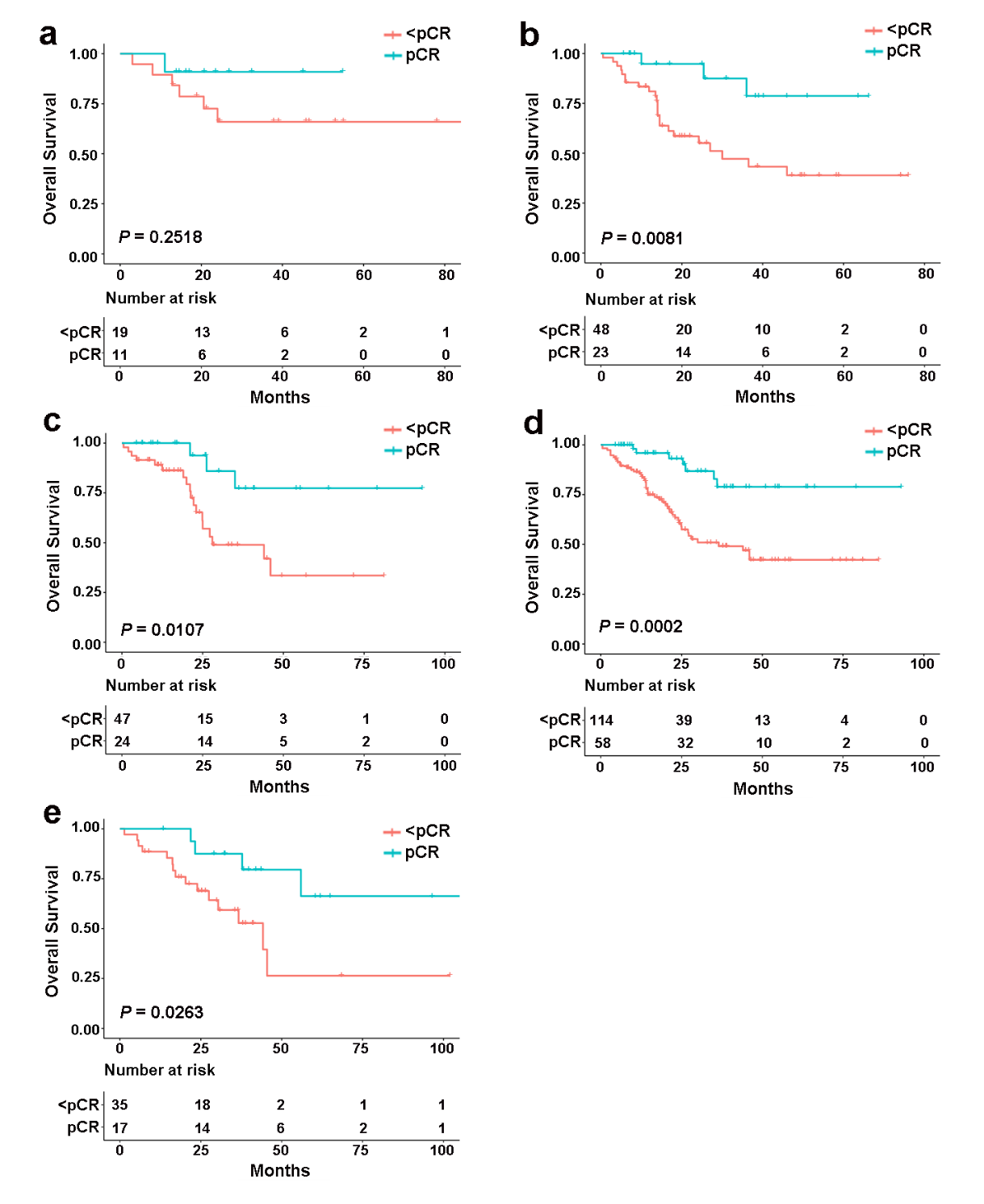
Figure. S1

Figure. S1. Overall survival of pCRs and <pCRs of esophageal squamous cell carcinoma after neoadjuvant chemoradiotherapy in different cohorts. A comparison of overall survival between pCRs and <pCRs in Beijing discovery cohort (a), Beijing training cohort (b), Beijing validation cohort (c), entire Beijing cohort (d), and external validation cohort (e).


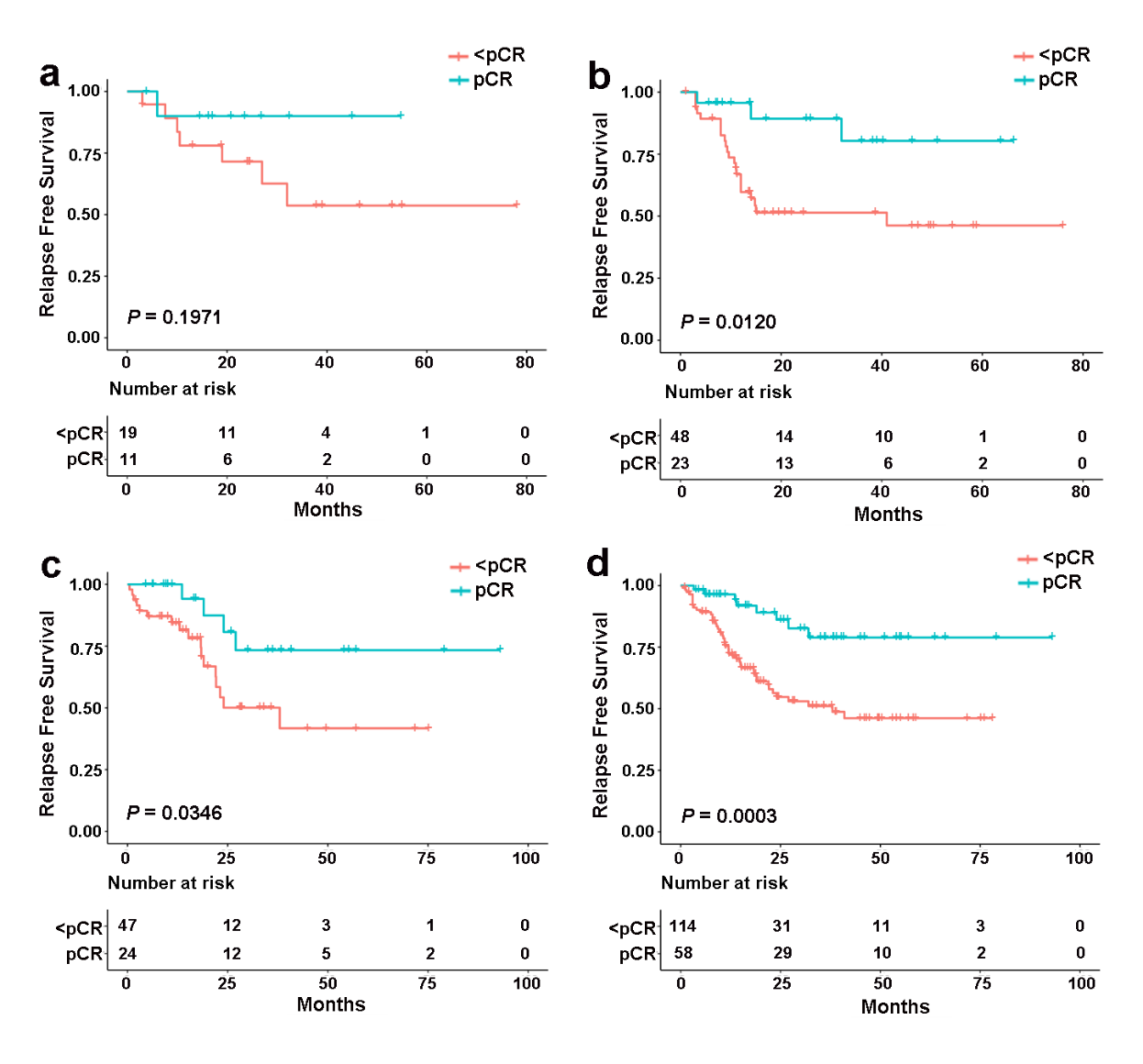
Figure. S2

Figure. S2. Relapse free survival of pCRs and <pCRs of esophageal squamous cell carcinoma after neoadjuvant chemoradiotherapy in Beijing cohort. A comparison of relapse free survival between pCRs and <pCRs in Beijing discovery cohort (a), Beijing training cohort (b), Beijing validation cohort (c) and entire Beijing cohort (d).


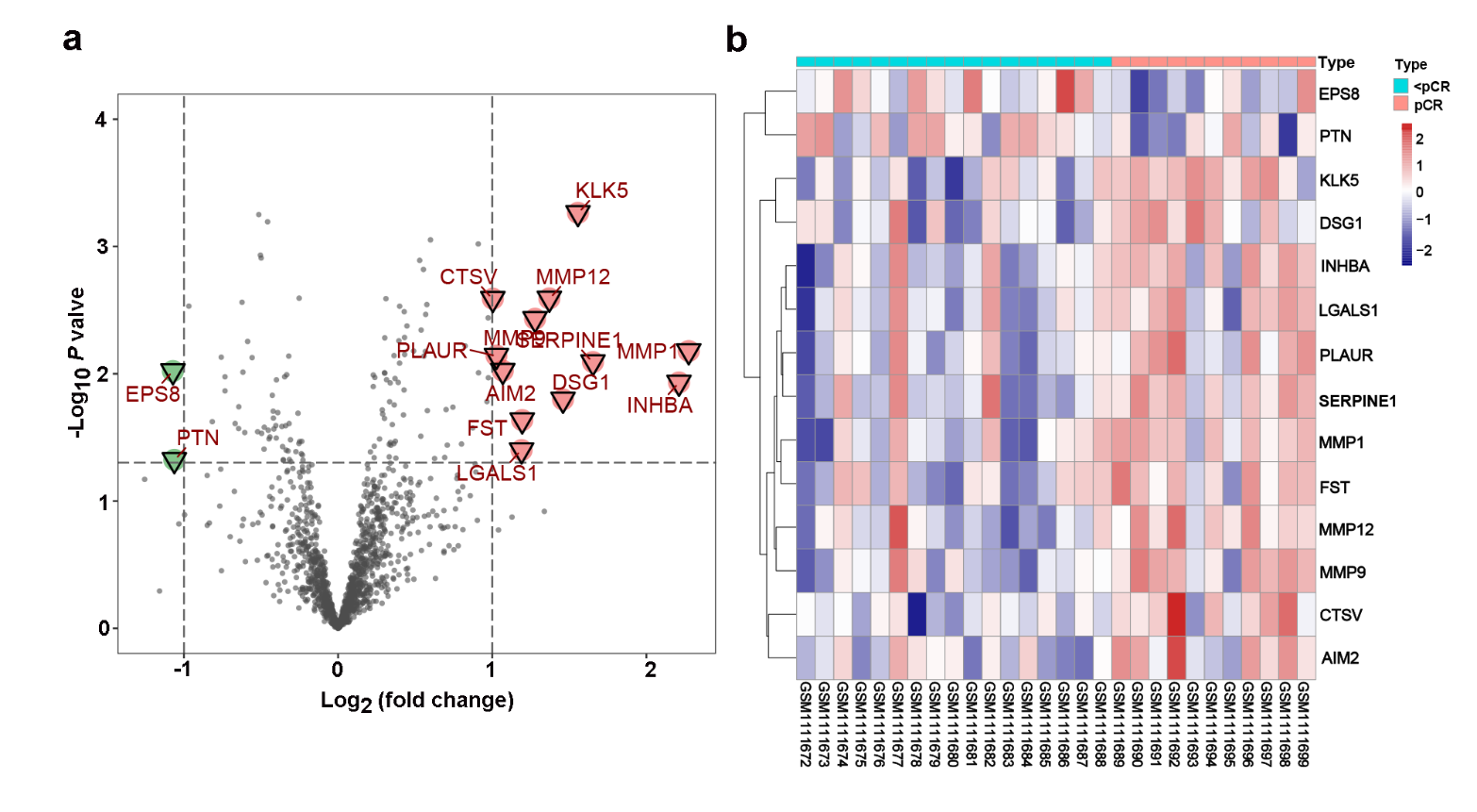
Figure. S3.

Figure. S3. Immune-related gene profiles from pretreatment biopsies between pCRs and <pCRs in Guangzhou cohort. a, volcano plot of differentially expressed immune-related genes with absolute log2 fold change over 1 and *P* value less than 0.05. b, a heatmap representing 14 differentially expressed immune-related genes between pCRs and <pCRs.


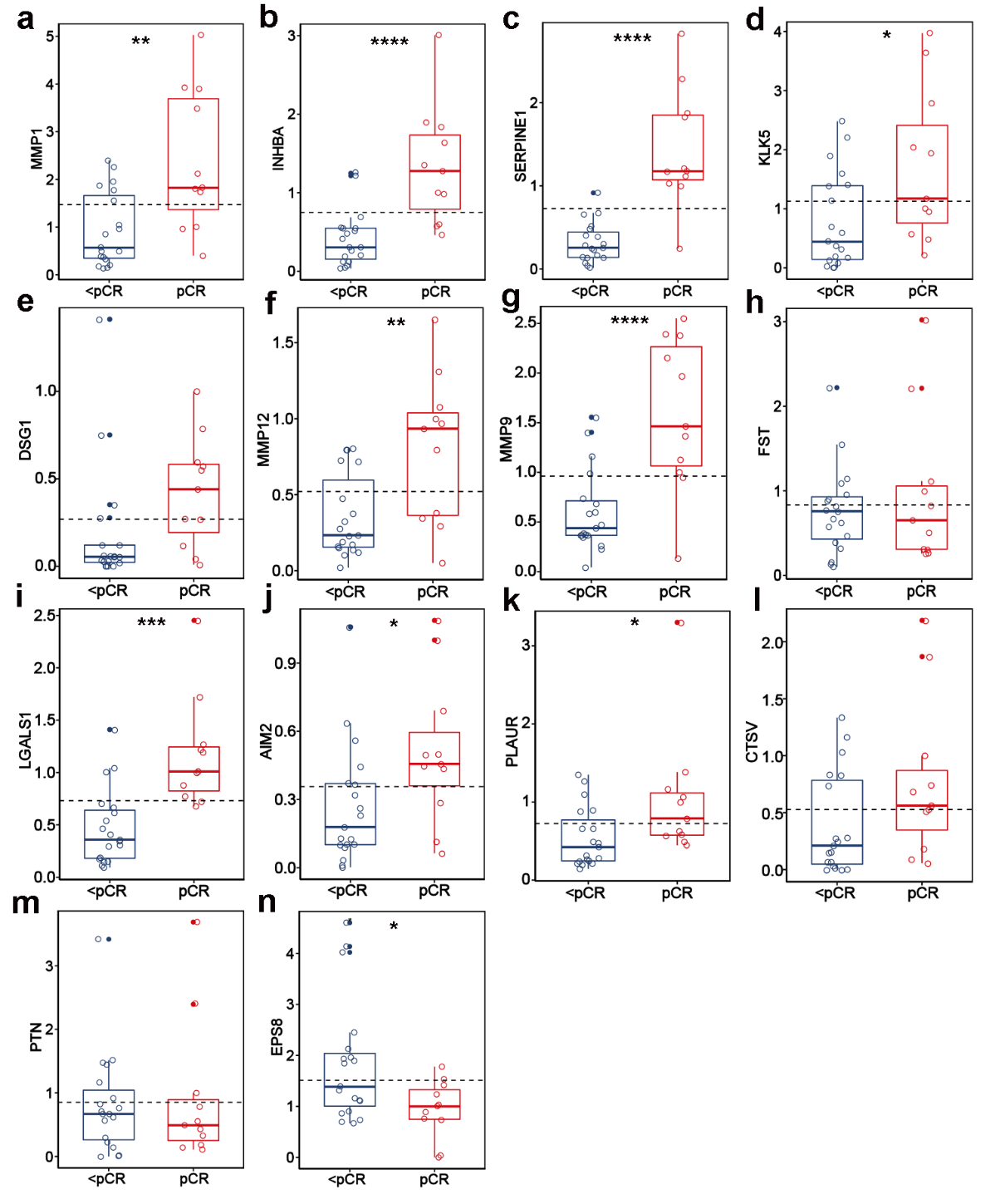
Figure. S4.

Figure. S4. The distributions of *MMP1* (a), *INHBA* (b), *SERPINE1* (c), *KLK5* (d), *DSG1* (e), *MMP12* (f), *MMP9* (g), *FST* (h), *LGALS1* (i) , *AIM2* (j), *PLAUR* (k), *CTSV* (l), *PTN* (m), and *EPS8* (n) expression levels in the Beijing discovery cohort determined by real-time quantitative polymerase chain reaction. *, **, ***, and **** represent *P* < 0.05, *P* < 0.01, *P* < 0.001, and *P* < 0.0001, respectively.


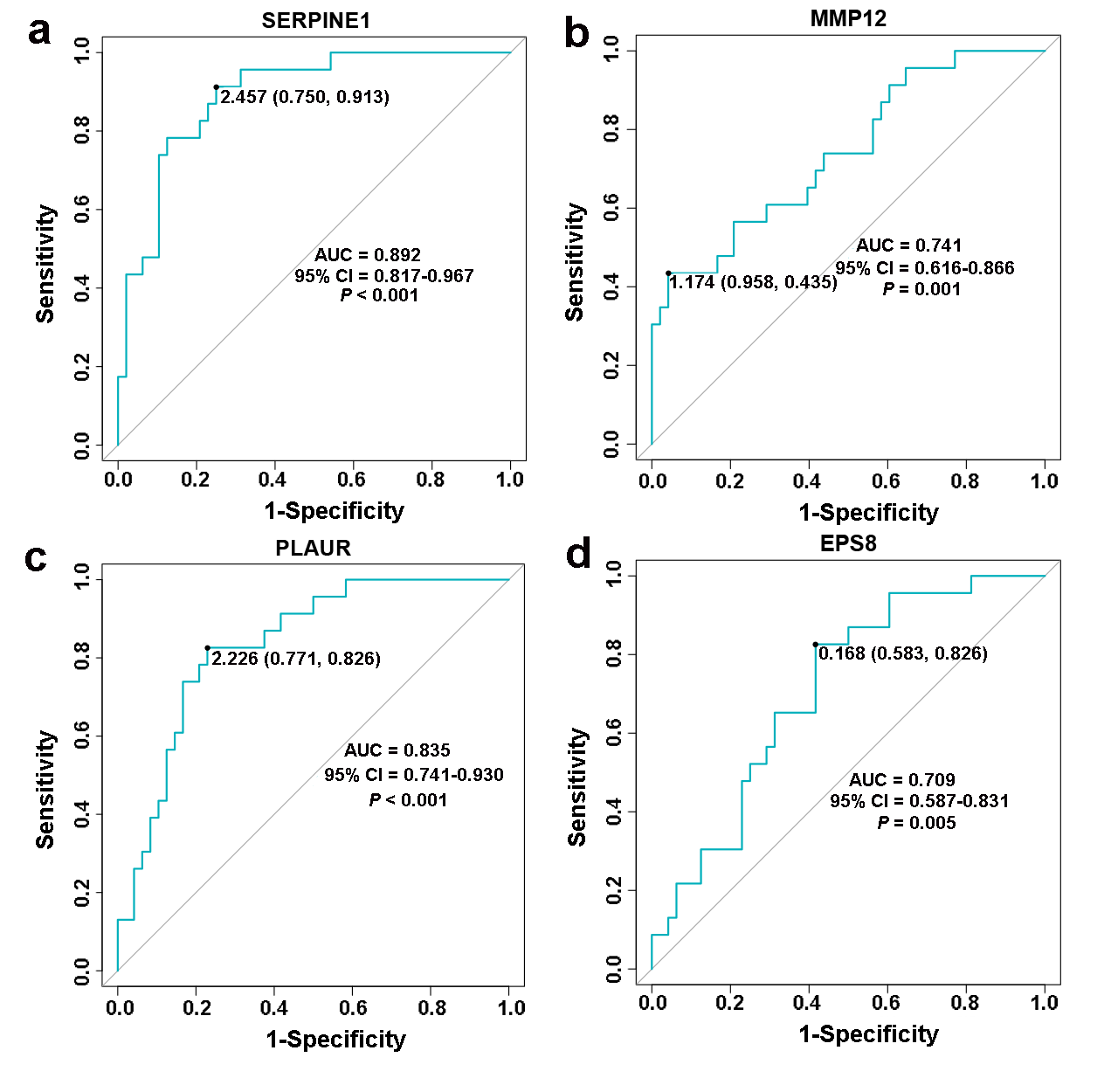
Figure. S5.

Figure. S5. Receiver operating characteristic curves of *SERPINE1* (a), *MMP12* (b), *PLAUR* (c), and *EPS8* (d) based on the real-time quantitative polymerase chain reaction value from Beijing training cohort.


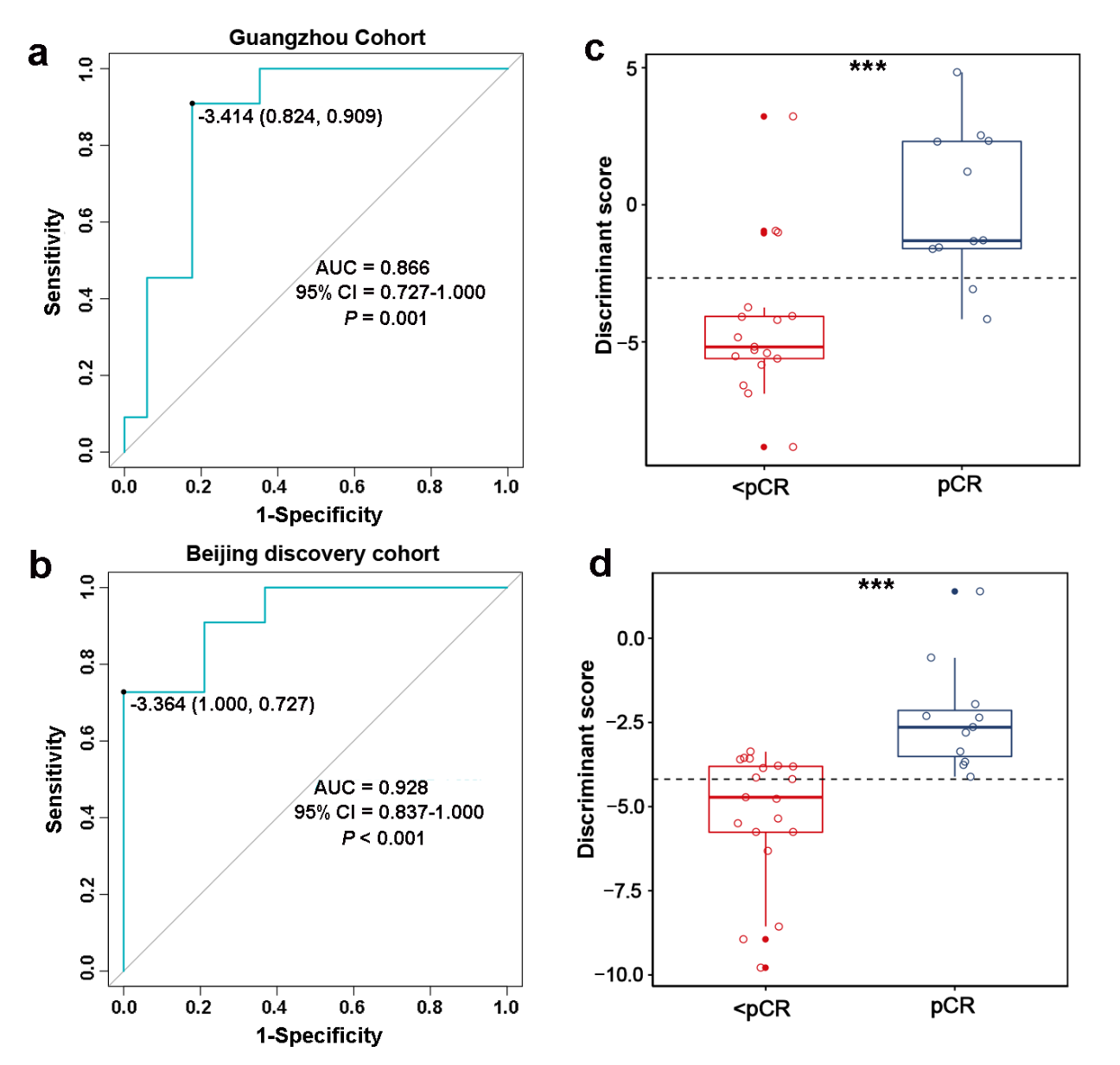
Figure. S6.

Figure. S6. Receiver operating characteristic curve (ROC) for the performance of the immune signature in Guangzhou cohort (a) and Beijing discovery cohort (b). Distributions of the discriminant scores between pCRs and <pCRs in Guangzhou cohort (c) and Beijing discovery cohort (d). *** represents *P* < 0.001.


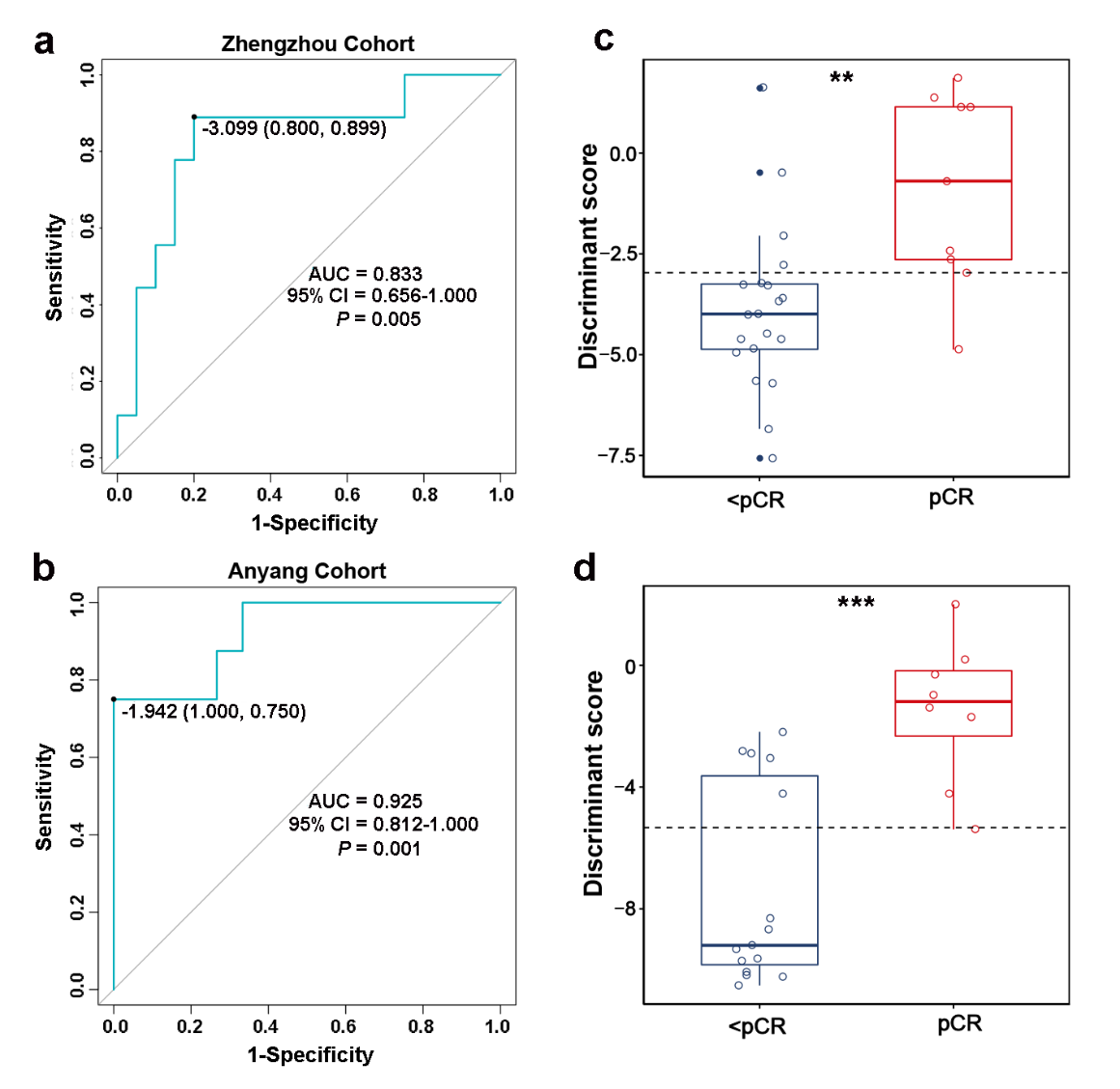
Figure. S7.

Figure. S7. Receiver operating characteristic curve (ROC) for the performance of the immune signature in Zhengzhou cohort (a) and Anyang cohort (b). Distributions of the discriminant scores between pCRs and <pCRs in Zhengzhou cohort (c) and Anyang cohort (d). * represents *P* < 0.05 and ** represents *P* < 0.01.


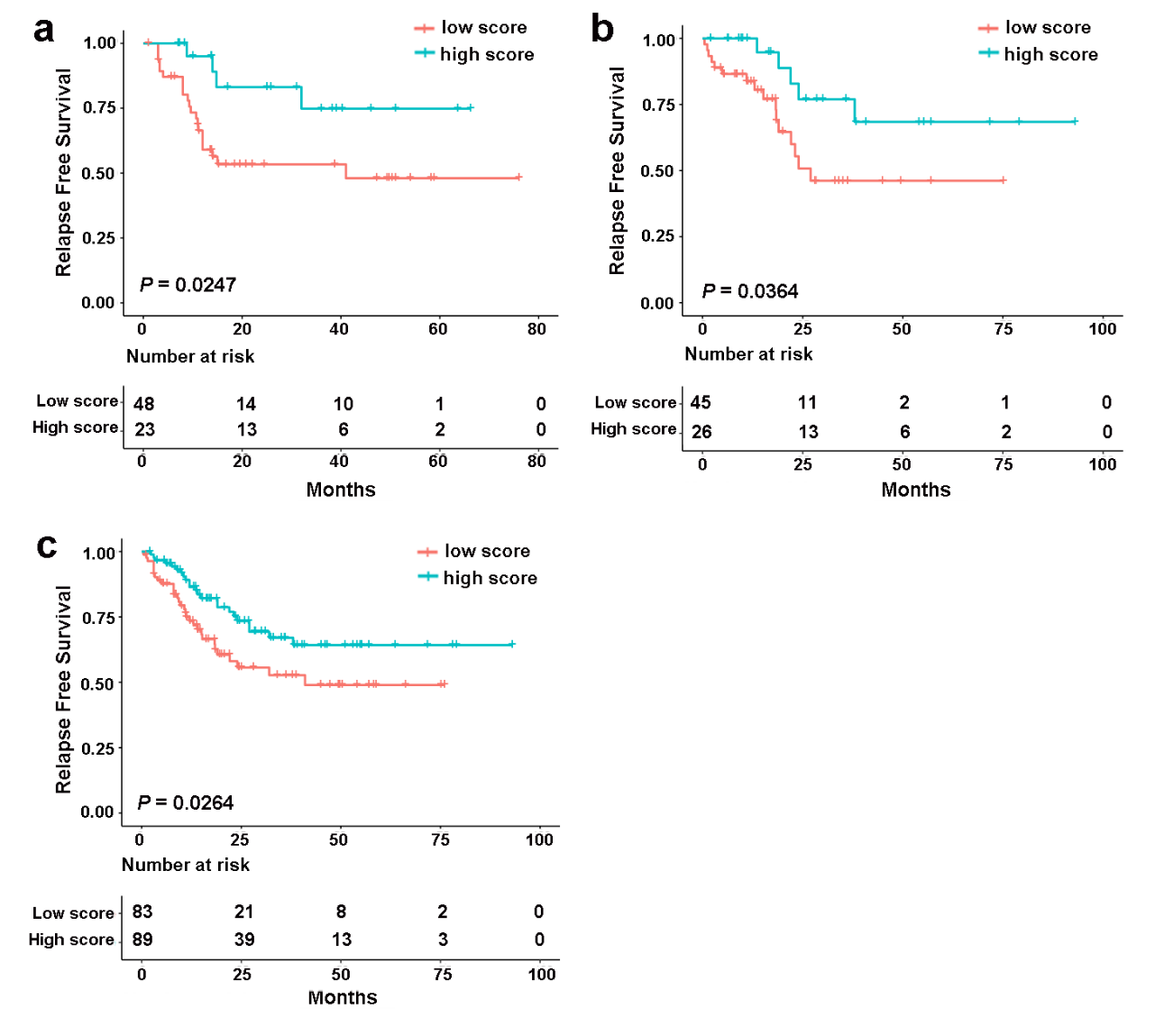
Figure. S8.

Figure. S8. Kaplan–Meier survival curves for relapse free survival (RFS) based on the discriminant scores of immune signature in training cohort (a), internal validation cohort (b) and entire Beijing cohort (c).

Table S1. The details of chemotherapy regimens in multiple institutions.

^a^, Platinum drugs/paclitaxel/nimotuzumab, platinum drugs/fluorouracil/nimotuzumab, or platinum drugs/paclitaxel/fluorouracil.

| chemotherapy regimens | Discovery cohort | | |  | Training cohort |  | Internal validation cohort |  | External validation cohort |
| --- | --- | --- | --- | --- | --- | --- | --- | --- | --- |
|  | Guangzhou cohort |  | Beijing  discovery cohort |  | Beijing  training cohort |  | Beijing  validation cohort |  | Integrated external validation cohort |
|  | (N=28) |  | (N=30) |  | (N=71) |  | (N=71) |  | (N=52) |
|  |  |  |  |  |  |  |  |  |  |
| Platinum drugs/vinorelbine | 28 |  | 0 |  | 0 |  | 0 |  | 0 |
| Platinum drugs/paclitaxel | 0 |  | 29 |  | 53 |  | 54 |  | 30 |
| Platinum drugs/fluorouracil | 0 |  | 1 |  | 12 |  | 13 |  | 22 |
| Platinum drugs/others^a^ | 0 |  | 0 |  | 6 |  | 4 |  | 0 |

Table S2. Primers for real-time quantitative polymerase chain reaction analysis.

| Gene Name | Forward Primer | Reverse Primer |
| --- | --- | --- |
| GAPDH | 5'-AACGACCACTTTGTCAAGC-3' | 5'-TGAGGTCCACCACCCTGT-3' |
| MMP1 | 5'-AGTACTGGGCTGTTCAGGGA-3' | 5'-TGTCCTTGGGGTATCCGTGT-3' |
| INHBA | 5'-AACTTTTGCTGCCAGGATGC-3' | 5'-GGTGGGGGAACTCCTCACTA-3' |
| SERPINE1 | 5'-GTGAGGGTGTTTCAGCAGGT-3' | 5'-GGGTGAGAAAACCACGTTGC-3' |
| KLK5 | 5'-CAGGCCAACTCCTGAGTCAT-3' | 5'-TGGGAAGGAATGAGGGTCTGA-3' |
| DSG1 | 5'-AGCCTGTCGTGAAGGTGAAG-3' | 5'-TCCTACTCCAGAGATGCGGT-3' |
| MMP12 | 5'-AGTTACCTTCAAAGGCCAAGAG-3' | 5'-AGTCCAAGGATGTTAGGAAGCA-3' |
| MMP9 | 5'-CGACGTCTTCCAGTACCGAG-3' | 5'-CTGGTTCAACTCACTCCGGG-3' |
| FST | 5'-CTGCTCCTCAGGTGTGCTAC-3' | 5'-TGTCTTCCGAAATGGAGTTGC-3' |
| LGALS1 | 5'-AAGCTGCCAGATGGATACGA-3' | 5'-CACCGTCAGCTGCCATGTAG-3' |
| AIM2 | 5'-AATAGCGCCTCACGTGTGTT-3' | 5'-TTCGGGGTTTCACCAGCTTT-3' |
| PLAUR | 5'-AGCCTTACCGAGGTTGTGTG-3' | 5'-AACGGCTTCGGGAATAGGTG-3' |
| CTSV | 5'-ACAACAACCCAGGCTCTAAGG-3' | 5'-TTCCGAGGGGTGCTGTTTTG-3' |
| PTN | 5'-GCAAGAAACAGGAGAAGATGC-3' | 5'-TGATCCTGTTTGCTGATGTCCT-3' |
| EPS8 | 5'-CGTCACCCAGGTGGATGTTA-3' | 5'-CTCAAAGTCCCCTTGGTCGG-3' |

Table S3. The list of 2695 immune-related genes enrolled in this study.

| No. | Gene symbol | No. | Gene symbol | No. | Gene symbol | No. | Gene symbol |
| --- | --- | --- | --- | --- | --- | --- | --- |
| 1 | THRA | 41 | DENND1B | 81 | NLRP9 | 121 | RPS6KA5 |
| 2 | CCL5 | 42 | CD200R1 | 82 | NLRP10 | 122 | HDAC4 |
| 3 | EPHB3 | 43 | TNFRSF13C | 83 | PIANP | 123 | DOCK2 |
| 4 | SCARB1 | 44 | GCSAML | 84 | EFNA2 | 124 | CANT1 |
| 5 | MAPK1 | 45 | IL23R | 85 | IFNE | 125 | STXBP4 |
| 6 | TMEM106A | 46 | CD276 | 86 | SPAG11A | 126 | FARP2 |
| 7 | CNOT7 | 47 | IFNL1 | 87 | MUCL1 | 127 | STAP1 |
| 8 | LEAP2 | 48 | TAB3 | 88 | FOXC1 | 128 | SIAE |
| 9 | SCIN | 49 | NLRP6 | 89 | PPARGC1B | 129 | NR1H4 |
| 10 | GAPT | 50 | ANGPT1 | 90 | PRF1 | 130 | TP73 |
| 11 | CLEC12A | 51 | CNPY3 | 91 | NLRX1 | 131 | PADI2 |
| 12 | DEFB106A | 52 | SCAMP1 | 92 | ADGRG3 | 132 | CST9 |
| 13 | IRF6 | 53 | SLC30A8 | 93 | IRAK2 | 133 | ACTR2 |
| 14 | PTPRC | 54 | IL27 | 94 | NKX2-3 | 134 | CLEC7A |
| 15 | CYP11B1 | 55 | DEFB125 | 95 | DCST1 | 135 | CTNNB1 |
| 16 | SLAMF6 | 56 | DEFB105A | 96 | MRGPRX2 | 136 | FBXO7 |
| 17 | CD300LG | 57 | TENM1 | 97 | ELMOD2 | 137 | DNAJB9 |
| 18 | HIPK1 | 58 | IL31RA | 98 | DCD | 138 | SAR1B |
| 19 | STON2 | 59 | CD300LF | 99 | EXOSC6 | 139 | SERPINC1 |
| 20 | CLEC4C | 60 | SERPINB12 | 100 | RAB7B | 140 | OSCAR |
| 21 | NLRC4 | 61 | PGLYRP3 | 101 | GPNMB | 141 | PIK3AP1 |
| 22 | IL12RB1 | 62 | BCL2L11 | 102 | MYO10 | 142 | CD80 |
| 23 | MMP21 | 63 | DEFB119 | 103 | ARSB | 143 | C2 |
| 24 | TMEM190 | 64 | RNF168 | 104 | ZBTB24 | 144 | RHOF |
| 25 | IFNL2 | 65 | PDE5A | 105 | SMPDL3B | 145 | MBP |
| 26 | JAK1 | 66 | SH2D1B | 106 | XG | 146 | ATP11B |
| 27 | ANLN | 67 | FCRL3 | 107 | DNAJA3 | 147 | APBB1IP |
| 28 | JMJD6 | 68 | BTNL9 | 108 | WDR78 | 148 | CYB5R3 |
| 29 | PTPN11 | 69 | ABCA13 | 109 | ZMYND11 | 149 | PSMD10 |
| 30 | TNFRSF10A | 70 | CSF3R | 110 | GOLGA7 | 150 | RUBCN |
| 31 | FLCN | 71 | CASP8 | 111 | CD300LB | 151 | SIRPB1 |
| 32 | LRRC7 | 72 | TNRC6A | 112 | PTPRO | 152 | OSBPL1A |
| 33 | ARL11 | 73 | HEATR9 | 113 | ITGAL | 153 | PDE4D |
| 34 | SLC4A1 | 74 | ATM | 114 | COCH | 154 | CD99L2 |
| 35 | HDAC9 | 75 | HFE | 115 | ERAP2 | 155 | ARHGEF2 |
| 36 | CLEC4D | 76 | AGBL4 | 116 | HAVCR2 | 156 | ADCY7 |
| 37 | TLR4 | 77 | SLFN13 | 117 | TRIM4 | 157 | PDGFRA |
| 38 | TIRAP | 78 | MUC19 | 118 | CYP19A1 | 158 | ELMO2 |
| 39 | SIGLEC10 | 79 | PAX1 | 119 | ITPKB | 159 | SDC3 |
| 40 | SENP1 | 80 | DEFB104A | 120 | SUPT6H | 160 | MITF |

| No. | Gene symbol | No. | Gene symbol | No. | Gene symbol | No. | Gene symbol |
| --- | --- | --- | --- | --- | --- | --- | --- |
| 161 | FCER1G | 201 | TREML1 | 241 | KMT2C | 281 | SAMHD1 |
| 162 | PDE12 | 202 | PTPRS | 242 | IRAK1BP1 | 282 | DNAJC13 |
| 163 | NLRP12 | 203 | SLA2 | 243 | DNASE1 | 283 | FCN1 |
| 164 | RAG1 | 204 | KLRK1 | 244 | ABCC9 | 284 | PRKCA |
| 165 | IL16 | 205 | CMTM3 | 245 | RAF1 | 285 | ITGA1 |
| 166 | IDH1 | 206 | CXADR | 246 | CD44 | 286 | CLTA |
| 167 | WNK1 | 207 | CD209 | 247 | HSP90AB1 | 287 | CALCOCO2 |
| 168 | STAT5B | 208 | BAP1 | 248 | SLC16A1 | 288 | ITGAD |
| 169 | APLF | 209 | LYZ | 249 | PLCG2 | 289 | DYRK3 |
| 170 | ATP6V0A2 | 210 | CD79B | 250 | EIF2B5 | 290 | CRACR2A |
| 171 | SLC11A1 | 211 | RBM15 | 251 | DNAJC3 | 291 | TNRC6C |
| 172 | CD96 | 212 | CD79A | 252 | MATR3 | 292 | XCR1 |
| 173 | OTUD7B | 213 | IRAK1 | 253 | TNRC6B | 293 | DNASE1L3 |
| 174 | C1S | 214 | ARPC5 | 254 | FN1 | 294 | ROCK1 |
| 175 | PGC | 215 | MOG | 255 | SRC | 295 | TAL1 |
| 176 | KLHL6 | 216 | CRISPLD2 | 256 | SRPK2 | 296 | BECN1 |
| 177 | ARMC8 | 217 | RHOA | 257 | SIRT2 | 297 | MUC4 |
| 178 | RAB24 | 218 | KLF6 | 258 | PECAM1 | 298 | CLECL1 |
| 179 | ADAM9 | 219 | RPS24 | 259 | VNN1 | 299 | NLRP1 |
| 180 | ITGA9 | 220 | PSMD6 | 260 | VPS35L | 300 | MARCH1 |
| 181 | ITGB2 | 221 | DNM2 | 261 | BANK1 | 301 | SLC8A3 |
| 182 | LRP1 | 222 | ADAM15 | 262 | FOXN1 | 302 | CLNK |
| 183 | ETS1 | 223 | DCTN5 | 263 | PIK3R6 | 303 | IST1 |
| 184 | ASAH1 | 224 | GON4L | 264 | AP2A1 | 304 | HBB |
| 185 | SYNCRIP | 225 | CD55 | 265 | THEMIS | 305 | CD200 |
| 186 | IFIH1 | 226 | NBEAL2 | 266 | FOXP1 | 306 | ITGAX |
| 187 | MCOLN2 | 227 | KDM6B | 267 | DHX36 | 307 | IRGM |
| 188 | IREB2 | 228 | RNASET2 | 268 | CGAS | 308 | RNF216 |
| 189 | IFNLR1 | 229 | TENT2 | 269 | PAK2 | 309 | DEFB107A |
| 190 | TYR | 230 | CAND1 | 270 | FNIP1 | 310 | SOX6 |
| 191 | TGFB3 | 231 | TRIM44 | 271 | CLEC4G | 311 | RUNX1 |
| 192 | TAPBP | 232 | COTL1 | 272 | FBXO9 | 312 | BTRC |
| 193 | KDELR1 | 233 | COL1A1 | 273 | OPRD1 | 313 | TREML4 |
| 194 | GATA1 | 234 | MYO1E | 274 | KAT6A | 314 | YTHDF3 |
| 195 | ZAP70 | 235 | SNAP25 | 275 | RAB6A | 315 | POLR3A |
| 196 | SLAMF1 | 236 | DYNC1H1 | 276 | KRAS | 316 | KLF13 |
| 197 | RAB34 | 237 | SERPINB6 | 277 | CLU | 317 | ESCO2 |
| 198 | LILRA5 | 238 | LEPR | 278 | LTBR | 318 | GSN |
| 199 | SAMSN1 | 239 | HGSNAT | 279 | MEIS1 | 319 | BTN2A2 |
| 200 | RBM14 | 240 | PLD1 | 280 | KMT2A | 320 | SLC9B2 |

| No. | Gene symbol | No. | Gene symbol | No. | Gene symbol | No. | Gene symbol |
| --- | --- | --- | --- | --- | --- | --- | --- |
| 321 | PRMT1 | 361 | CAPZA2 | 401 | NDRG1 | 441 | EPRS |
| 322 | MGST1 | 362 | SPON2 | 402 | UBB | 442 | PRDX6 |
| 323 | TFE3 | 363 | LILRB4 | 403 | SOD1 | 443 | FLNA |
| 324 | RARA | 364 | EREG | 404 | CALM1 | 444 | PSAP |
| 325 | LRRK1 | 365 | DACT2 | 405 | PHB | 445 | CCT8 |
| 326 | MUC6 | 366 | POU2AF1 | 406 | S100A11 | 446 | PSMB1 |
| 327 | ARPC3 | 367 | MYSM1 | 407 | CTSA | 447 | EPAS1 |
| 328 | ALDH3B1 | 368 | ATP8A1 | 408 | CD63 | 448 | PSMD4 |
| 329 | N4BP2L2 | 369 | STK4 | 409 | UBE2D3 | 449 | STAT1 |
| 330 | PAK1 | 370 | ATG7 | 410 | CD81 | 450 | AHCY |
| 331 | IKZF1 | 371 | DYNC1I1 | 411 | GRN | 451 | HLA-E |
| 332 | TAB2 | 372 | FGL1 | 412 | GLO1 | 452 | SLC3A2 |
| 333 | PLD4 | 373 | STX8 | 413 | HSPA9 | 453 | RAB14 |
| 334 | CD6 | 374 | STXBP2 | 414 | HK1 | 454 | VCL |
| 335 | PDXK | 375 | GAS6 | 415 | NPC2 | 455 | DCTN2 |
| 336 | NFE2L2 | 376 | MMP14 | 416 | DYNLL1 | 456 | CALR |
| 337 | ZNF160 | 377 | BAD | 417 | SKP1 | 457 | SEC31A |
| 338 | RAC1 | 378 | PARK7 | 418 | RPL13A | 458 | ARPC1A |
| 339 | CD74 | 379 | SRP14 | 419 | ACTR1A | 459 | SDCBP |
| 340 | DEFB124 | 380 | GDI2 | 420 | CAPN1 | 460 | CD59 |
| 341 | PIK3R2 | 381 | PSMB2 | 421 | NFE2L1 | 461 | SERPING1 |
| 342 | IDO2 | 382 | SART1 | 422 | CTSD | 462 | PSME3 |
| 343 | IRAK3 | 383 | ILF2 | 423 | GNAS | 463 | HIF1A |
| 344 | ACKR2 | 384 | RPL30 | 424 | PSMB7 | 464 | TRIM28 |
| 345 | FANCD2 | 385 | ARF1 | 425 | IQGAP1 | 465 | IPO7 |
| 346 | INPP5D | 386 | CANX | 426 | XRCC6 | 466 | ACTR3 |
| 347 | SPN | 387 | KARS | 427 | HSPA1A | 467 | CKAP4 |
| 348 | NUB1 | 388 | RPS6 | 428 | ACTB | 468 | CD9 |
| 349 | MSH6 | 389 | EEF2 | 429 | TMBIM6 | 469 | ANXA1 |
| 350 | IGF2R | 390 | HSP90B1 | 430 | HSPD1 | 470 | JUP |
| 351 | MIA3 | 391 | MSN | 431 | ZYX | 471 | CD99 |
| 352 | TP53BP1 | 392 | APP | 432 | PAFAH1B1 | 472 | DUSP1 |
| 353 | SOX13 | 393 | PRKAR1A | 433 | PSME1 | 473 | PSMF1 |
| 354 | CXCL2 | 394 | DSP | 434 | PSMD8 | 474 | MYL9 |
| 355 | PIK3C3 | 395 | WDR1 | 435 | LAMP2 | 475 | STOM |
| 356 | GLI3 | 396 | AP2B1 | 436 | GSTP1 | 476 | PABPC4 |
| 357 | ALCAM | 397 | AP2M1 | 437 | PSMD2 | 477 | PSMC2 |
| 358 | TET2 | 398 | CLTC | 438 | RAP1B | 478 | PFKL |
| 359 | FLG2 | 399 | MLEC | 439 | BCAP31 | 479 | LGALS1 |
| 360 | PAFAH1B2 | 400 | CAP1 | 440 | CTSB | 480 | THBS1 |

| No. | Gene symbol | No. | Gene symbol | No. | Gene symbol | No. | Gene symbol |
| --- | --- | --- | --- | --- | --- | --- | --- |
| 481 | PSMA7 | 521 | COPB1 | 561 | CNN2 | 601 | RNF41 |
| 482 | PGRMC1 | 522 | CST3 | 562 | PRDX3 | 602 | PGM1 |
| 483 | ITGB5 | 523 | ZFP36L2 | 563 | NBL1 | 603 | KIF5B |
| 484 | ACLY | 524 | PSMD3 | 564 | EIF2B1 | 604 | ST6GAL1 |
| 485 | PJA2 | 525 | ITGA5 | 565 | CLPTM1 | 605 | DYNLT1 |
| 486 | HLA-DPB1 | 526 | PSMB3 | 566 | BST2 | 606 | LTF |
| 487 | RAB5C | 527 | SOX4 | 567 | IFNGR2 | 607 | ALDOC |
| 488 | HAX1 | 528 | IFI30 | 568 | TSTA3 | 608 | ACAA1 |
| 489 | PUM1 | 529 | CUL4A | 569 | CNIH1 | 609 | SFRP1 |
| 490 | HTRA1 | 530 | VIM | 570 | ITGA6 | 610 | MYO18A |
| 491 | SLC7A5 | 531 | CAT | 571 | GJA1 | 611 | CTR9 |
| 492 | PSMD1 | 532 | GBF1 | 572 | USP14 | 612 | LDLR |
| 493 | CREG1 | 533 | ATP6AP2 | 573 | DLG5 | 613 | SDC4 |
| 494 | CSTB | 534 | MAPKAPK2 | 574 | TPD52 | 614 | OPTN |
| 495 | TNFAIP1 | 535 | JUN | 575 | EGR1 | 615 | BIRC2 |
| 496 | HDAC1 | 536 | SHC1 | 576 | PNP | 616 | SEC14L1 |
| 497 | DDX3X | 537 | JUNB | 577 | PSMC6 | 617 | MX1 |
| 498 | LGMN | 538 | PYGB | 578 | CCND3 | 618 | CTSL |
| 499 | ARIH2 | 539 | QSOX1 | 579 | PSMD7 | 619 | PAF1 |
| 500 | PSMD13 | 540 | CTSC | 580 | ACIN1 | 620 | IGBP1 |
| 501 | DDX1 | 541 | PUM2 | 581 | CD14 | 621 | DPF2 |
| 502 | ATP1B1 | 542 | PRCP | 582 | TP53 | 622 | CPNE3 |
| 503 | OTUB1 | 543 | NFKBIA | 583 | PSME2 | 623 | AP2S1 |
| 504 | PKM | 544 | G3BP1 | 584 | RELA | 624 | ABL1 |
| 505 | PSMC4 | 545 | UBE2N | 585 | ADAR | 625 | RIOK3 |
| 506 | BAG6 | 546 | APOD | 586 | LBR | 626 | ACTR1B |
| 507 | PSMC3 | 547 | ZFP36 | 587 | LPCAT1 | 627 | CREBBP |
| 508 | NME2 | 548 | PSMA3 | 588 | EPCAM | 628 | PKN1 |
| 509 | PSMA5 | 549 | DUSP3 | 589 | BNIP3 | 629 | PRKCZ |
| 510 | RAB5B | 550 | ACTG1 | 590 | COL3A1 | 630 | MVP |
| 511 | APEH | 551 | LAMP1 | 591 | ABCE1 | 631 | KAT2A |
| 512 | SDC1 | 552 | GYG1 | 592 | B4GALT1 | 632 | KIF22 |
| 513 | TOP2A | 553 | VAMP2 | 593 | CEACAM5 | 633 | SRPK1 |
| 514 | PRNP | 554 | ID2 | 594 | B2M | 634 | EP300 |
| 515 | IFITM2 | 555 | GLB1 | 595 | IMPDH2 | 635 | CRK |
| 516 | PSMA2 | 556 | NME1 | 596 | PRDX4 | 636 | TRIB1 |
| 517 | STAT6 | 557 | SFPQ | 597 | HEXB | 637 | PSMB4 |
| 518 | VAMP3 | 558 | ANXA2 | 598 | CCT2 | 638 | STXBP1 |
| 519 | UBE2D2 | 559 | INPPL1 | 599 | CAPZB | 639 | BMI1 |
| 520 | FLOT2 | 560 | IFITM1 | 600 | CIB1 | 640 | GBP1 |

| No. | Gene symbol | No. | Gene symbol | No. | Gene symbol | No. | Gene symbol |
| --- | --- | --- | --- | --- | --- | --- | --- |
| 641 | PDGFRB | 681 | IRF1 | 721 | CASP3 | 761 | SLPI |
| 642 | G6PD | 682 | FADD | 722 | FBN1 | 762 | CYBA |
| 643 | CDKN1A | 683 | AIMP1 | 723 | STK39 | 763 | PTPRN2 |
| 644 | MTOR | 684 | PRKCD | 724 | MAPKAPK3 | 764 | PIAS3 |
| 645 | PDAP1 | 685 | VAMP8 | 725 | PLCG1 | 765 | STK10 |
| 646 | CTSH | 686 | GGT1 | 726 | SEC24B | 766 | TCTA |
| 647 | TAP1 | 687 | HSPA1B | 727 | PRKACA | 767 | CAV1 |
| 648 | BCR | 688 | S100A13 | 728 | TOM1 | 768 | CTNNBIP1 |
| 649 | CSK | 689 | ADAM10 | 729 | GAA | 769 | KIF2A |
| 650 | UNG | 690 | GUSB | 730 | HEXIM1 | 770 | CSF1R |
| 651 | UBE2K | 691 | EPS8 | 731 | AHR | 771 | PTK2B |
| 652 | ITGAV | 692 | CTPS1 | 732 | VAMP7 | 772 | SLC11A2 |
| 653 | PSMD12 | 693 | IRF3 | 733 | SERPINA1 | 773 | RB1 |
| 654 | SEC24C | 694 | LYN | 734 | TRAFD1 | 774 | DAPK1 |
| 655 | RAP1A | 695 | SERPINE1 | 735 | FUCA1 | 775 | BCL6 |
| 656 | CBFB | 696 | ICAM1 | 736 | HPRT1 | 776 | AP3B1 |
| 657 | SEC24D | 697 | TNFAIP3 | 737 | SLC16A3 | 777 | TRIM14 |
| 658 | KLF10 | 698 | NRAS | 738 | CXCL8 | 778 | NECTIN2 |
| 659 | ABCF3 | 699 | RPS19 | 739 | SP100 | 779 | IFIT1 |
| 660 | SRF | 700 | MARCH7 | 740 | OAS1 | 780 | RNF8 |
| 661 | COL1A2 | 701 | WIPF1 | 741 | CD93 | 781 | TIMP2 |
| 662 | IGF2 | 702 | EFNB2 | 742 | DDIT4 | 782 | RHOG |
| 663 | IFI27 | 703 | MAP2K1 | 743 | ANPEP | 783 | RASSF2 |
| 664 | DHX9 | 704 | AXL | 744 | SIRPA | 784 | DOCK1 |
| 665 | PPP3CA | 705 | TNFSF10 | 745 | CTSS | 785 | ESRRA |
| 666 | PLSCR1 | 706 | OXSR1 | 746 | NBN | 786 | MAPK9 |
| 667 | MYC | 707 | NUDT21 | 747 | S100A8 | 787 | APRT |
| 668 | PPP3CB | 708 | TMEM63A | 748 | YES1 | 788 | IL4R |
| 669 | NOTCH2 | 709 | TRIM26 | 749 | SOX9 | 789 | LGALS9 |
| 670 | CTSK | 710 | CCNB2 | 750 | ZMPSTE24 | 790 | DCTN6 |
| 671 | HDAC5 | 711 | HIST2H2BE | 751 | GYPC | 791 | MAP2K4 |
| 672 | EIF2B2 | 712 | EFNB1 | 752 | IL1R1 | 792 | TUSC2 |
| 673 | ELP1 | 713 | PTPN1 | 753 | C1QB | 793 | IRF2 |
| 674 | PPIE | 714 | IGFBP2 | 754 | HCLS1 | 794 | HLA-DQA1 |
| 675 | SLC2A3 | 715 | IFNGR1 | 755 | KIF13B | 795 | CNOT4 |
| 676 | TRIM29 | 716 | PRKACB | 756 | MPP1 | 796 | JARID2 |
| 677 | ATG5 | 717 | ITM2A | 757 | TRAF3IP2 | 797 | AP1S2 |
| 678 | DLG1 | 718 | GBP2 | 758 | RGS1 | 798 | GNL1 |
| 679 | MLH1 | 719 | SLC7A8 | 759 | PYGL | 799 | STXBP3 |
| 680 | MAPK14 | 720 | GPC1 | 760 | C7 | 800 | NCK2 |

| No. | Gene symbol | No. | Gene symbol | No. | Gene symbol | No. | Gene symbol |
| --- | --- | --- | --- | --- | --- | --- | --- |
| 801 | SH2B3 | 841 | RAB4A | 881 | KAT2B | 921 | P2RX4 |
| 802 | IDE | 842 | DYNC1LI2 | 882 | TRIM32 | 922 | CCL4 |
| 803 | KIFAP3 | 843 | FSTL3 | 883 | AKAP8 | 923 | IL2RG |
| 804 | AP1G1 | 844 | IFIT5 | 884 | GAB2 | 924 | CD48 |
| 805 | APOE | 845 | POLB | 885 | CFI | 925 | TYROBP |
| 806 | ARRB2 | 846 | AKAP17A | 886 | ADARB1 | 926 | FOXO3 |
| 807 | KIF3C | 847 | SKP2 | 887 | VCAM1 | 927 | ZP3 |
| 808 | HES1 | 848 | IGF1R | 888 | PIK3CD | 928 | MFNG |
| 809 | PSMA4 | 849 | GPRC5B | 889 | RAB11FIP2 | 929 | TCIRG1 |
| 810 | KCNAB2 | 850 | MID1 | 890 | THBD | 930 | ENPP4 |
| 811 | SATB1 | 851 | FGFR2 | 891 | DAPK3 | 931 | EMILIN1 |
| 812 | CD53 | 852 | PLA2G2A | 892 | CRCP | 932 | SBNO2 |
| 813 | KMT2B | 853 | CTSF | 893 | TAB1 | 933 | IMPDH1 |
| 814 | HEBP2 | 854 | TRIM13 | 894 | DNASE1L1 | 934 | MB |
| 815 | MME | 855 | HMOX1 | 895 | CXCL9 | 935 | RARG |
| 816 | PSMD5 | 856 | CXCL12 | 896 | NR1H3 | 936 | IFNAR1 |
| 817 | LDB1 | 857 | DGAT1 | 897 | CYBB | 937 | PKNOX1 |
| 818 | PSEN1 | 858 | GNS | 898 | MAPT | 938 | RUNX3 |
| 819 | CHD2 | 859 | TARBP2 | 899 | HLA-DMB | 939 | PDGFB |
| 820 | PLEK | 860 | VEGFB | 900 | KDR | 940 | CEBPG |
| 821 | IQGAP2 | 861 | BCL2 | 901 | MMP9 | 941 | APOBEC3G |
| 822 | OTUD4 | 862 | CX3CL1 | 902 | KIF3B | 942 | EIF2AK2 |
| 823 | ELF4 | 863 | PI3 | 903 | BTN2A1 | 943 | PIGR |
| 824 | LRRC14 | 864 | FZD7 | 904 | ARG2 | 944 | RAB32 |
| 825 | MED1 | 865 | PDE4B | 905 | MPO | 945 | PSMC1 |
| 826 | EPHA2 | 866 | DPP4 | 906 | NMI | 946 | GMFG |
| 827 | BPGM | 867 | ERCC1 | 907 | CEBPD | 947 | GLIPR1 |
| 828 | TNFRSF1B | 868 | BAK1 | 908 | TAZ | 948 | GCH1 |
| 829 | LYST | 869 | HMGB3 | 909 | CASP9 | 949 | FLI1 |
| 830 | SEMA4D | 870 | AQP3 | 910 | PAWR | 950 | DCTN3 |
| 831 | PPP6C | 871 | CEACAM6 | 911 | FCGR3B | 951 | LMO2 |
| 832 | STX4 | 872 | GCA | 912 | PURA | 952 | GPSM3 |
| 833 | S100A9 | 873 | MANBA | 913 | PDCD2 | 953 | EDNRB |
| 834 | CD4 | 874 | SEMA3C | 914 | PCBP2 | 954 | PSMB9 |
| 835 | AOC1 | 875 | FANCA | 915 | SCG2 | 955 | PMAIP1 |
| 836 | GGH | 876 | ELF2 | 916 | CEBPA | 956 | SYNGR1 |
| 837 | AGL | 877 | IL32 | 917 | IRF8 | 957 | STK11 |
| 838 | TRIM38 | 878 | LRRC32 | 918 | PRKX | 958 | LOX |
| 839 | NFIL3 | 879 | MAP3K5 | 919 | THOC1 | 959 | CD151 |
| 840 | SLC7A6 | 880 | RPS6KA3 | 920 | STK3 | 960 | ATP1B2 |

| No. | Gene symbol | No. | Gene symbol | No. | Gene symbol | No. | Gene symbol |
| --- | --- | --- | --- | --- | --- | --- | --- |
| 961 | CREB1 | 1001 | REST | 1041 | MLF1 | 1081 | CENPE |
| 962 | NF1 | 1002 | RAPGEF1 | 1042 | IFNAR2 | 1082 | KIT |
| 963 | TMC6 | 1003 | STAR | 1043 | VSIG4 | 1083 | EXOSC9 |
| 964 | AGA | 1004 | IKBKE | 1044 | SMAD7 | 1084 | ARID4A |
| 965 | SEC23A | 1005 | AHSG | 1045 | EML1 | 1085 | ENPP1 |
| 966 | S100P | 1006 | IRF4 | 1046 | MYB | 1086 | IL1B |
| 967 | LIMK1 | 1007 | SELL | 1047 | TRIM21 | 1087 | XRCC4 |
| 968 | SKAP2 | 1008 | MMP12 | 1048 | HLA-F | 1088 | NCAPH2 |
| 969 | SP2 | 1009 | CD22 | 1049 | BTN3A3 | 1089 | ZBTB1 |
| 970 | PIK3CA | 1010 | KLK3 | 1050 | MELK | 1090 | ATP6V0A1 |
| 971 | DCAF1 | 1011 | L1CAM | 1051 | FGL2 | 1091 | CCR1 |
| 972 | KYNU | 1012 | SLC7A7 | 1052 | APAF1 | 1092 | CIITA |
| 973 | ACPP | 1013 | VPS33A | 1053 | NAIP | 1093 | EFNA4 |
| 974 | KCNN4 | 1014 | EXO1 | 1054 | IL6ST | 1094 | APOB |
| 975 | RHBDD3 | 1015 | CCL21 | 1055 | PDPN | 1095 | FPR1 |
| 976 | SLC12A2 | 1016 | ITGB3 | 1056 | LCK | 1096 | NCF4 |
| 977 | TRAF2 | 1017 | MYH2 | 1057 | PTGER4 | 1097 | TRIL |
| 978 | IFI6 | 1018 | COL17A1 | 1058 | BCL3 | 1098 | CD40 |
| 979 | SLC2A5 | 1019 | ADA | 1059 | SOX11 | 1099 | SPTBN2 |
| 980 | PLEKHO2 | 1020 | S1PR1 | 1060 | TLR2 | 1100 | MYLPF |
| 981 | MRC1 | 1021 | HLA-DRB1 | 1061 | INHBA | 1101 | RBBP5 |
| 982 | IFI44L | 1022 | MUC2 | 1062 | TCF21 | 1102 | STAT2 |
| 983 | CD83 | 1023 | LRMP | 1063 | PTPN2 | 1103 | CD58 |
| 984 | ARSA | 1024 | ICAM2 | 1064 | MAP4K2 | 1104 | QPCT |
| 985 | KIF11 | 1025 | HHEX | 1065 | FST | 1105 | ADAM8 |
| 986 | ALOX5 | 1026 | PLA2G6 | 1066 | ICAM3 | 1106 | SPINK5 |
| 987 | APOA1 | 1027 | CHGA | 1067 | RHOH | 1107 | SMAD5 |
| 988 | SNCA | 1028 | ISG20 | 1068 | MNDA | 1108 | FANCC |
| 989 | PTPRZ1 | 1029 | KIF23 | 1069 | OAS2 | 1109 | MAP3K14 |
| 990 | CXCL1 | 1030 | NCK1 | 1070 | RAB3A | 1110 | AP1S1 |
| 991 | GEM | 1031 | TGFBR3 | 1071 | EMP2 | 1111 | ATP7A |
| 992 | MMP1 | 1032 | TRIM23 | 1072 | FGB | 1112 | RELB |
| 993 | OSTF1 | 1033 | MT1G | 1073 | MX2 | 1113 | IL6 |
| 994 | CCN3 | 1034 | IFIT3 | 1074 | ADORA2A | 1114 | ACVR1B |
| 995 | DMTN | 1035 | NR1D1 | 1075 | MAP3K8 | 1115 | POLR3F |
| 996 | PPP3R1 | 1036 | ARHGEF5 | 1076 | EFNB3 | 1116 | ESR1 |
| 997 | ELMO1 | 1037 | TAP2 | 1077 | ITGA2 | 1117 | IL1RAP |
| 998 | PDPK1 | 1038 | THBS4 | 1078 | DEFA3 | 1118 | CXCL13 |
| 999 | TOX | 1039 | HOXB7 | 1079 | ORM1 | 1119 | NOTCH4 |
| 1000 | CXCL10 | 1040 | FAS | 1080 | ORM2 | 1120 | CEP290 |

| No. | Gene symbol | No. | Gene symbol | No. | Gene symbol | No. | Gene symbol |
| --- | --- | --- | --- | --- | --- | --- | --- |
| 1121 | PBX1 | 1161 | GNLY | 1201 | CD8A | 1241 | BATF |
| 1122 | TCF7 | 1162 | ZNF175 | 1202 | SLC27A2 | 1242 | CD1C |
| 1123 | BCL10 | 1163 | C5 | 1203 | F12 | 1243 | CD84 |
| 1124 | LIF | 1164 | BTK | 1204 | KLK7 | 1244 | WNT5A |
| 1125 | ADD2 | 1165 | GCNT1 | 1205 | ITGAM | 1245 | IL15 |
| 1126 | LCP2 | 1166 | TCN1 | 1206 | CD1D | 1246 | PRG4 |
| 1127 | GTPBP1 | 1167 | VAV2 | 1207 | SKAP1 | 1247 | CASP1 |
| 1128 | LRP8 | 1168 | CR2 | 1208 | IL7R | 1248 | SOCS6 |
| 1129 | FYB1 | 1169 | TYK2 | 1209 | WASL | 1249 | TNFAIP6 |
| 1130 | BAIAP2 | 1170 | BPI | 1210 | REG3A | 1250 | MERTK |
| 1131 | SPI1 | 1171 | TRAF6 | 1211 | SIX1 | 1251 | RAB33A |
| 1132 | KIF5A | 1172 | LAMP3 | 1212 | MARCO | 1252 | SELP |
| 1133 | ACVR2A | 1173 | PIP4K2A | 1213 | CD2 | 1253 | PTPN22 |
| 1134 | SNX4 | 1174 | ANGPT2 | 1214 | GYPA | 1254 | PPIL2 |
| 1135 | S100A1 | 1175 | ROR2 | 1215 | JAK2 | 1255 | ADGRB1 |
| 1136 | HOXB6 | 1176 | HRH1 | 1216 | PTPRB | 1256 | PRKCH |
| 1137 | SH2B2 | 1177 | ETV6 | 1217 | ZBTB7B | 1257 | RNASE2 |
| 1138 | LRRC17 | 1178 | RASGRP1 | 1218 | LY86 | 1258 | EGR3 |
| 1139 | CFD | 1179 | TNFSF12 | 1219 | S100A12 | 1259 | CD33 |
| 1140 | CCL14 | 1180 | MEOX1 | 1220 | FCN3 | 1260 | CXCR5 |
| 1141 | SMAD3 | 1181 | CDA | 1221 | TREX1 | 1261 | XAF1 |
| 1142 | WAS | 1182 | FGA | 1222 | RET | 1262 | ADAMDEC1 |
| 1143 | PRSS2 | 1183 | CTSG | 1223 | PRKD1 | 1263 | FZD5 |
| 1144 | IL1R2 | 1184 | C4BPA | 1224 | ZBTB16 | 1264 | RHAG |
| 1145 | FES | 1185 | OASL | 1225 | ITGA4 | 1265 | CD27 |
| 1146 | GPR183 | 1186 | LY75 | 1226 | REG1B | 1266 | PTX3 |
| 1147 | AP1B1 | 1187 | CYP27B1 | 1227 | MSH3 | 1267 | IL13RA2 |
| 1148 | TBKBP1 | 1188 | CD86 | 1228 | ADORA2B | 1268 | BMP6 |
| 1149 | BMP5 | 1189 | CD38 | 1229 | CX3CR1 | 1269 | ARG1 |
| 1150 | MST1R | 1190 | MAP2K6 | 1230 | KRT1 | 1270 | CRKL |
| 1151 | CD3E | 1191 | IL17RA | 1231 | MICA | 1271 | GPR17 |
| 1152 | RAB35 | 1192 | TRPM2 | 1232 | MICB | 1272 | GRB14 |
| 1153 | IRF5 | 1193 | BST1 | 1233 | FOXJ1 | 1273 | CD180 |
| 1154 | CCL20 | 1194 | ITGB7 | 1234 | S100A7 | 1274 | CLC |
| 1155 | AMBP | 1195 | PKP1 | 1235 | RAB3B | 1275 | SELE |
| 1156 | PLAU | 1196 | SCGB1A1 | 1236 | IL27RA | 1276 | WNT10B |
| 1157 | ADORA1 | 1197 | ADAM17 | 1237 | CTSE | 1277 | PLA2G7 |
| 1158 | ISG15 | 1198 | SH3GL2 | 1238 | FOXF1 | 1278 | EDA |
| 1159 | SIT1 | 1199 | CRP | 1239 | HK3 | 1279 | VAV1 |
| 1160 | GZMA | 1200 | F2 | 1240 | IL6R | 1280 | HRG |

| No. | Gene symbol | No. | Gene symbol | No. | Gene symbol | No. | Gene symbol |
| --- | --- | --- | --- | --- | --- | --- | --- |
| 1281 | LIG4 | 1321 | F2RL1 | 1361 | SKIL | 1401 | NDUFC2 |
| 1282 | CR1 | 1322 | S1PR4 | 1362 | CEACAM8 | 1402 | SPTA1 |
| 1283 | PRKCE | 1323 | SEMG1 | 1363 | CD5L | 1403 | POU4F1 |
| 1284 | BLK | 1324 | PDE1B | 1364 | CLEC10A | 1404 | TGFBR1 |
| 1285 | CPN1 | 1325 | MASP1 | 1365 | PTPN6 | 1405 | BGLAP |
| 1286 | GPLD1 | 1326 | DBH | 1366 | IL7 | 1406 | CXCR6 |
| 1287 | TLR3 | 1327 | WNT2B | 1367 | HP | 1407 | LTA |
| 1288 | LY6D | 1328 | DHRS2 | 1368 | TEK | 1408 | HSPH1 |
| 1289 | PTAFR | 1329 | BMX | 1369 | RIPOR2 | 1409 | CCR2 |
| 1290 | ADCYAP1 | 1330 | CD5 | 1370 | UMOD | 1410 | C8B |
| 1291 | IL18 | 1331 | LAG3 | 1371 | LMO1 | 1411 | CCR5 |
| 1292 | TEC | 1332 | CD36 | 1372 | C9 | 1412 | TSC22D3 |
| 1293 | C8A | 1333 | ITGA2B | 1373 | CHRNA4 | 1413 | CXCR2 |
| 1294 | PLA2G1B | 1334 | PML | 1374 | VEGFD | 1414 | KITLG |
| 1295 | HLA-DOA | 1335 | CD70 | 1375 | CD1B | 1415 | NKX3-2 |
| 1296 | EPPIN | 1336 | PIP | 1376 | EDN2 | 1416 | GLI2 |
| 1297 | RFX1 | 1337 | AIM2 | 1377 | FCER2 | 1417 | TNFRSF11A |
| 1298 | DAPK2 | 1338 | MGAM | 1378 | KLRC1 | 1418 | MASP2 |
| 1299 | IFI16 | 1339 | CD28 | 1379 | HTN3 | 1419 | KRT75 |
| 1300 | GALNS | 1340 | TAC1 | 1380 | PAX5 | 1420 | SMAD6 |
| 1301 | CXCL6 | 1341 | CEACAM1 | 1381 | CD3G | 1421 | IL18RAP |
| 1302 | CCR7 | 1342 | NKX2-5 | 1382 | L3MBTL1 | 1422 | NLRP3 |
| 1303 | CARTPT | 1343 | LY96 | 1383 | TXK | 1423 | ASS1 |
| 1304 | IL2RA | 1344 | CNR2 | 1384 | HOXA7 | 1424 | CSF1 |
| 1305 | PRLR | 1345 | GFI1 | 1385 | RNASE3 | 1425 | P2RX7 |
| 1306 | APCS | 1346 | DRD2 | 1386 | MAP3K7 | 1426 | LEP |
| 1307 | SOCS3 | 1347 | AP3D1 | 1387 | HYAL2 | 1427 | CXCR1 |
| 1308 | XCL1 | 1348 | INS | 1388 | LILRB5 | 1428 | LILRB1 |
| 1309 | XCL2 | 1349 | ENDOU | 1389 | FKBP1B | 1429 | ADGRE1 |
| 1310 | CPLX2 | 1350 | CBL | 1390 | ELANE | 1430 | TNF |
| 1311 | PIK3CG | 1351 | IL18R1 | 1391 | APOA4 | 1431 | GGT2 |
| 1312 | FOLR3 | 1352 | APOBEC3B | 1392 | ICAM5 | 1432 | ALPK1 |
| 1313 | G3BP2 | 1353 | CHRNB2 | 1393 | TNFSF9 | 1433 | CDK6 |
| 1314 | PF4 | 1354 | HTN1 | 1394 | CFHR2 | 1434 | CITED1 |
| 1315 | CD19 | 1355 | TNFRSF17 | 1395 | TRIM25 | 1435 | MSTN |
| 1316 | NPFF | 1356 | DSG1 | 1396 | FOXE1 | 1436 | GLMN |
| 1317 | CCL13 | 1357 | CPB2 | 1397 | CRTAM | 1437 | APOBEC1 |
| 1318 | FER | 1358 | POLR3G | 1398 | CPNE1 | 1438 | IL12A |
| 1319 | RORC | 1359 | IGLL1 | 1399 | IL11 | 1439 | AKT1 |
| 1320 | IGSF6 | 1360 | FLT3 | 1400 | CH25H | 1440 | CD101 |

| No. | Gene symbol | No. | Gene symbol | No. | Gene symbol | No. | Gene symbol |
| --- | --- | --- | --- | --- | --- | --- | --- |
| 1441 | ADIPOQ | 1481 | TLR6 | 1521 | FCAR | 1561 | IL3 |
| 1442 | FRK | 1482 | GYPB | 1522 | ARTN | 1562 | TNFSF14 |
| 1443 | JAK3 | 1483 | GZMM | 1523 | CXCR3 | 1563 | MAEA |
| 1444 | ICAM4 | 1484 | PRSS3 | 1524 | LILRB2 | 1564 | IFNA8 |
| 1445 | TNIP1 | 1485 | PIR | 1525 | SEC13 | 1565 | CNR1 |
| 1446 | TNFSF8 | 1486 | SNRK | 1526 | RBCK1 | 1566 | IL5 |
| 1447 | PTBP3 | 1487 | MEIS2 | 1527 | POU4F2 | 1567 | PRKCB |
| 1448 | SIGLEC7 | 1488 | BTN3A1 | 1528 | LAX1 | 1568 | IFNA10 |
| 1449 | PRKACG | 1489 | MS4A2 | 1529 | RNF125 | 1569 | GLG1 |
| 1450 | MBL2 | 1490 | LAIR2 | 1530 | NCKAP1 | 1570 | MEF2C |
| 1451 | EPO | 1491 | BDKRB1 | 1531 | METTL7A | 1571 | NR4A3 |
| 1452 | DEFA4 | 1492 | CHRNB4 | 1532 | PQBP1 | 1572 | CD8B2 |
| 1453 | CD300C | 1493 | RO60 | 1533 | RBPJ | 1573 | CITED2 |
| 1454 | MMP25 | 1494 | IL1RL1 | 1534 | KLRD1 | 1574 | GNRH1 |
| 1455 | F7 | 1495 | SLC7A11 | 1535 | CRISP3 | 1575 | ARPC2 |
| 1456 | KIR3DL2 | 1496 | DEFA5 | 1536 | FCN2 | 1576 | OPRM1 |
| 1457 | CD226 | 1497 | CDKN2B | 1537 | PSMD9 | 1577 | AMPD3 |
| 1458 | CDK13 | 1498 | CCL1 | 1538 | PROS1 | 1578 | CLEC4M |
| 1459 | DSC1 | 1499 | NFKB2 | 1539 | ATP6AP1 | 1579 | HCK |
| 1460 | ALOX15 | 1500 | IL4 | 1540 | DEFA6 | 1580 | CACNA1C |
| 1461 | MMP8 | 1501 | SYK | 1541 | PF4V1 | 1581 | ADD1 |
| 1462 | TFRC | 1502 | CD46 | 1542 | IFNW1 | 1582 | MADCAM1 |
| 1463 | TGFBR2 | 1503 | OPRK1 | 1543 | PTK2 | 1583 | IL1RL2 |
| 1464 | LTB | 1504 | PITX2 | 1544 | AIF1 | 1584 | HIST1H4A |
| 1465 | PRTN3 | 1505 | MR1 | 1545 | CD160 | 1585 | CEACAM3 |
| 1466 | CCL16 | 1506 | THEMIS2 | 1546 | IL13 | 1586 | CBFA2T3 |
| 1467 | DEFB4A | 1507 | SHH | 1547 | POU1F1 | 1587 | CCR8 |
| 1468 | PGLYRP1 | 1508 | ADGRE2 | 1548 | MUC1 | 1588 | LAIR1 |
| 1469 | CYP7B1 | 1509 | CAMK2A | 1549 | IL2 | 1589 | CCL7 |
| 1470 | GP1BA | 1510 | CUL1 | 1550 | CXCL3 | 1590 | ZBP1 |
| 1471 | BTN1A1 | 1511 | TANK | 1551 | LILRA2 | 1591 | CFHR5 |
| 1472 | NPY5R | 1512 | SLC7A2 | 1552 | NCR1 | 1592 | AIRE |
| 1473 | NFATC3 | 1513 | PDCD1 | 1553 | CCL22 | 1593 | SOCS5 |
| 1474 | RAC2 | 1514 | FZD9 | 1554 | LILRA1 | 1594 | DDX17 |
| 1475 | COLEC10 | 1515 | TNFRSF13B | 1555 | CFHR4 | 1595 | DDX21 |
| 1476 | TNFSF4 | 1516 | GPR171 | 1556 | CD40LG | 1596 | CHIT1 |
| 1477 | DEGS1 | 1517 | CMKLR1 | 1557 | CCL17 | 1597 | TRIM31 |
| 1478 | IL10 | 1518 | BLNK | 1558 | IL12B | 1598 | IFNB1 |
| 1479 | CSF3 | 1519 | SH3PXD2A | 1559 | IL5RA | 1599 | KIR2DL1 |
| 1480 | CCR9 | 1520 | TBX1 | 1560 | LNPEP | 1600 | IFNA16 |

| No. | Gene symbol | No. | Gene symbol | No. | Gene symbol | No. | Gene symbol |
| --- | --- | --- | --- | --- | --- | --- | --- |
| 1601 | IL9 | 1641 | CD1E | 1681 | THY1 | 1721 | NFKB1 |
| 1602 | NFATC1 | 1642 | LILRB3 | 1682 | LCP1 | 1722 | METTL3 |
| 1603 | C4BPB | 1643 | HUWE1 | 1683 | HLA-DRA | 1723 | PRKD2 |
| 1604 | DMBT1 | 1644 | HOXA3 | 1684 | ATP6V1D | 1724 | TNFRSF10B |
| 1605 | PSG1 | 1645 | NTRK1 | 1685 | C1QBP | 1725 | PPM1B |
| 1606 | IFNA7 | 1646 | WNT4 | 1686 | CYFIP1 | 1726 | CA2 |
| 1607 | MEFV | 1647 | SAA1 | 1687 | NEU1 | 1727 | SWAP70 |
| 1608 | HMHB1 | 1648 | SPTAN1 | 1688 | ILF3 | 1728 | AZGP1 |
| 1609 | CRLF2 | 1649 | PDIA3 | 1689 | LGALS3 | 1729 | CASP4 |
| 1610 | CCR3 | 1650 | FLNB | 1690 | ERP44 | 1730 | IKBKB |
| 1611 | GPI | 1651 | EZR | 1691 | KPNB1 | 1731 | TNFRSF14 |
| 1612 | MALT1 | 1652 | VAT1 | 1692 | TUBB4B | 1732 | ECM1 |
| 1613 | TRAF3 | 1653 | ACTN1 | 1693 | CRIP2 | 1733 | ANXA3 |
| 1614 | IFNA1 | 1654 | XRCC5 | 1694 | NCOA6 | 1734 | POLR3C |
| 1615 | POLR3D | 1655 | PARP1 | 1695 | UBC | 1735 | TSC1 |
| 1616 | BRCA2 | 1656 | RPS14 | 1696 | TCF12 | 1736 | ENPP2 |
| 1617 | CAPZA1 | 1657 | VCP | 1697 | STAT3 | 1737 | CHI3L1 |
| 1618 | CCR4 | 1658 | CD24 | 1698 | ABI1 | 1738 | FAM3A |
| 1619 | CACNA1F | 1659 | DDOST | 1699 | CADM1 | 1739 | KIF2C |
| 1620 | SGPL1 | 1660 | PA2G4 | 1700 | MDK | 1740 | IFI35 |
| 1621 | MID2 | 1661 | BSG | 1701 | PSMB8 | 1741 | THOC5 |
| 1622 | EDN3 | 1662 | PRDX1 | 1702 | NSD2 | 1742 | MSH2 |
| 1623 | IL17A | 1663 | HSPA8 | 1703 | CORO1A | 1743 | EIF2B4 |
| 1624 | CD164 | 1664 | RPS3 | 1704 | JAG1 | 1744 | PATZ1 |
| 1625 | GRAP2 | 1665 | PRKDC | 1705 | UFD1 | 1745 | CREB3 |
| 1626 | HOXB3 | 1666 | RPL39 | 1706 | TSPAN6 | 1746 | FBXW11 |
| 1627 | CIAPIN1 | 1667 | CDC42 | 1707 | MYD88 | 1747 | PTN |
| 1628 | KIR2DL4 | 1668 | HLA-B | 1708 | KRT6A | 1748 | LRP5 |
| 1629 | MECOM | 1669 | FLOT1 | 1709 | SNAP23 | 1749 | HLA-DQB1 |
| 1630 | IRF7 | 1670 | NCSTN | 1710 | PRKRA | 1750 | RARRES2 |
| 1631 | FGR | 1671 | SUMO1 | 1711 | TCF3 | 1751 | TNFSF13 |
| 1632 | NECTIN1 | 1672 | LTA4H | 1712 | MAN2B1 | 1752 | PSMC5 |
| 1633 | RAB3D | 1673 | ANKHD1 | 1713 | IRS2 | 1753 | RAB27A |
| 1634 | BAX | 1674 | PSMD11 | 1714 | FOS | 1754 | ASH2L |
| 1635 | PPARG | 1675 | VAPA | 1715 | DIAPH1 | 1755 | CACNB3 |
| 1636 | GPR15 | 1676 | PSMB5 | 1716 | PIM1 | 1756 | IGF1 |
| 1637 | IFNA6 | 1677 | PSMA6 | 1717 | CXCR4 | 1757 | CD34 |
| 1638 | WNT1 | 1678 | HMGB2 | 1718 | LMO4 | 1758 | RIPK2 |
| 1639 | HIST1H3A | 1679 | PSMB6 | 1719 | SEC22B | 1759 | APOL1 |
| 1640 | HIST1H2BK | 1680 | ATP1B3 | 1720 | GPC3 | 1760 | APOBEC3C |

| No. | Gene symbol | No. | Gene symbol | No. | Gene symbol | No. | Gene symbol |
| --- | --- | --- | --- | --- | --- | --- | --- |
| 1761 | GATA3 | 1801 | MAP2K7 | 1841 | GPR18 | 1881 | NCR3 |
| 1762 | PLD2 | 1802 | CDC37 | 1842 | FLT1 | 1882 | FPR2 |
| 1763 | PGF | 1803 | CAMK2B | 1843 | KLRG1 | 1883 | ARNT |
| 1764 | TTR | 1804 | NPPA | 1844 | LILRA4 | 1884 | PLAUR |
| 1765 | CHUK | 1805 | NCAM1 | 1845 | C8G | 1885 | FASLG |
| 1766 | S100B | 1806 | NFKBIL1 | 1846 | CD1A | 1886 | APOBEC3A |
| 1767 | GATA2 | 1807 | SOCS1 | 1847 | TPO | 1887 | ZEB1 |
| 1768 | SERPINB3 | 1808 | OLR1 | 1848 | CAMK4 | 1888 | FCGR2B |
| 1769 | SERPINB9 | 1809 | HPX | 1849 | IFNG | 1889 | ERBB2 |
| 1770 | GM2A | 1810 | IDO1 | 1850 | MS4A1 | 1890 | LEF1 |
| 1771 | HLA-DRB4 | 1811 | CD247 | 1851 | LY9 | 1891 | RTN4 |
| 1772 | CLEC2B | 1812 | NOS2 | 1852 | CCL15 | 1892 | CD47 |
| 1773 | NCKAP1L | 1813 | PRKCQ | 1853 | DEFB1 | 1893 | CXCL11 |
| 1774 | ITCH | 1814 | PGM3 | 1854 | P2RX1 | 1894 | IFNA21 |
| 1775 | ACE | 1815 | CTSZ | 1855 | SERPINB4 | 1895 | THPO |
| 1776 | REG1A | 1816 | LYL1 | 1856 | RORA | 1896 | PSTPIP1 |
| 1777 | MFAP5 | 1817 | AQP4 | 1857 | ICOS | 1897 | ICOSLG |
| 1778 | TMEM131L | 1818 | CCL19 | 1858 | MAPK8 | 1898 | IFNA2 |
| 1779 | JAG2 | 1819 | CTSV | 1859 | KLF1 | 1899 | ITK |
| 1780 | ERAP1 | 1820 | AGER | 1860 | FUT7 | 1900 | KIR3DL1 |
| 1781 | KRT16 | 1821 | SEMA7A | 1861 | VEGFA | 1901 | PADI4 |
| 1782 | IL33 | 1822 | FYN | 1862 | HLA-G | 1902 | TYRO3 |
| 1783 | DNASE2 | 1823 | SH2D1A | 1863 | HNF1A | 1903 | CCRL2 |
| 1784 | BTN3A2 | 1824 | IL1A | 1864 | BIRC3 | 1904 | BMP4 |
| 1785 | CDH17 | 1825 | LTB4R | 1865 | TRIM27 | 1905 | ALAS2 |
| 1786 | CDC42EP2 | 1826 | CCL11 | 1866 | CCL23 | 1906 | LBP |
| 1787 | SPHK2 | 1827 | CST7 | 1867 | SIGLEC9 | 1907 | POU2F2 |
| 1788 | SELPLG | 1828 | INHA | 1868 | TRIM10 | 1908 | DYNC1I2 |
| 1789 | SLIT2 | 1829 | GZMB | 1869 | LST1 | 1909 | TUBB |
| 1790 | HOXA9 | 1830 | TLR5 | 1870 | GPER1 | 1910 | FCER1A |
| 1791 | C3AR1 | 1831 | C6 | 1871 | TNFSF11 | 1911 | EVI2B |
| 1792 | TGFB2 | 1832 | PTPRJ | 1872 | TNFRSF10D | 1912 | UBE2D1 |
| 1793 | DHTKD1 | 1833 | TLR1 | 1873 | AGPAT2 | 1913 | LAT2 |
| 1794 | CCL18 | 1834 | TRIM15 | 1874 | SPPL2B | 1914 | SERINC3 |
| 1795 | NFE2 | 1835 | FKBP1A | 1875 | TRIM5 | 1915 | AP2A2 |
| 1796 | CD300A | 1836 | GABPA | 1876 | ADGRE3 | 1916 | DCTN1 |
| 1797 | PARP3 | 1837 | HSP90AA1 | 1877 | CALCA | 1917 | KMT2D |
| 1798 | RIPK1 | 1838 | CSF2 | 1878 | ONECUT1 | 1918 | MPL |
| 1799 | VEGFC | 1839 | CAMP | 1879 | EPHB1 | 1919 | CFB |
| 1800 | NCF2 | 1840 | MS4A3 | 1880 | GRB7 | 1920 | PLCB1 |

| No. | Gene symbol | No. | Gene symbol | No. | Gene symbol | No. | Gene symbol |
| --- | --- | --- | --- | --- | --- | --- | --- |
| 1921 | MYH9 | 1961 | RRAS | 2001 | NFASC | 2041 | KLRB1 |
| 1922 | PRRC2C | 1962 | IL1RN | 2002 | MFHAS1 | 2042 | FSHB |
| 1923 | ITGB1 | 1963 | PVR | 2003 | ERCC2 | 2043 | CEBPE |
| 1924 | RAB7A | 1964 | TIPARP | 2004 | COL2A1 | 2044 | SERPINB10 |
| 1925 | ZFP36L1 | 1965 | CAMK2G | 2005 | CD3D | 2045 | FPR3 |
| 1926 | HLA-DPA1 | 1966 | ZNF3 | 2006 | RNASE6 | 2046 | IFNA5 |
| 1927 | PXDN | 1967 | PIK3CB | 2007 | BMPR1A | 2047 | AZU1 |
| 1928 | MAPK3 | 1968 | PHLPP1 | 2008 | SIPA1L3 | 2048 | TNFRSF21 |
| 1929 | WIPF2 | 1969 | PIK3R4 | 2009 | KIF3A | 2049 | TSPAN2 |
| 1930 | C1R | 1970 | OLFM4 | 2010 | C12orf29 | 2050 | PAK3 |
| 1931 | MTUS1 | 1971 | SOS1 | 2011 | SECTM1 | 2051 | MNX1 |
| 1932 | KPNA6 | 1972 | FTL | 2012 | GPS2 | 2052 | EPX |
| 1933 | RBFOX2 | 1973 | SERINC5 | 2013 | HIPK2 | 2053 | MYO1C |
| 1934 | FAF2 | 1974 | JAM3 | 2014 | RSAD2 | 2054 | MUC3A |
| 1935 | ZMIZ1 | 1975 | PAXIP1 | 2015 | CFH | 2055 | RIF1 |
| 1936 | LSM14A | 1976 | GRAMD4 | 2016 | CACTIN | 2056 | CDC42EP4 |
| 1937 | SDC2 | 1977 | ARHGAP45 | 2017 | HOXA5 | 2057 | SBSPON |
| 1938 | MT2A | 1978 | KLC1 | 2018 | RAP2B | 2058 | MAP3K1 |
| 1939 | IFITM3 | 1979 | WDR7 | 2019 | HLA-A | 2059 | TRIM56 |
| 1940 | ANKRD17 | 1980 | ABR | 2020 | CCL8 | 2060 | LRP5L |
| 1941 | FASN | 1981 | SEC24A | 2021 | RPL22 | 2061 | KAT8 |
| 1942 | PSME4 | 1982 | ADGRF5 | 2022 | CD7 | 2062 | LRCH1 |
| 1943 | ASXL1 | 1983 | ACKR3 | 2023 | DOK2 | 2063 | CXCL5 |
| 1944 | PIK3R1 | 1984 | HRAS | 2024 | IFI44 | 2064 | APOBEC3F |
| 1945 | SERPINB1 | 1985 | MAPK8IP1 | 2025 | SHMT2 | 2065 | TRIM58 |
| 1946 | MCM3AP | 1986 | RNF19B | 2026 | CLDN18 | 2066 | PRDX2 |
| 1947 | PSMD14 | 1987 | ZC3HAV1 | 2027 | PPBP | 2067 | GRB2 |
| 1948 | TXLNA | 1988 | ARID5A | 2028 | ITFG2 | 2068 | ADTRP |
| 1949 | SCAP | 1989 | SP3 | 2029 | SFTPD | 2069 | RAG2 |
| 1950 | ELF1 | 1990 | CDKN1C | 2030 | FTH1 | 2070 | DOCK10 |
| 1951 | SETD2 | 1991 | TICAM1 | 2031 | TNFRSF4 | 2071 | HLA-DRB3 |
| 1952 | CEBPB | 1992 | SETD1A | 2032 | GAL | 2072 | LFNG |
| 1953 | PICALM | 1993 | PIBF1 | 2033 | MUC5AC | 2073 | CD8B |
| 1954 | PIP5K1C | 1994 | SARM1 | 2034 | MAPK10 | 2074 | CFHR1 |
| 1955 | LCN2 | 1995 | TRIM22 | 2035 | GLA | 2075 | STOML2 |
| 1956 | SCRIB | 1996 | CYLD | 2036 | HLX | 2076 | IHH |
| 1957 | GAPDH | 1997 | ZBTB7A | 2037 | CTSW | 2077 | DUSP10 |
| 1958 | JCHAIN | 1998 | PLCL2 | 2038 | HIST1H2BC | 2078 | ETV2 |
| 1959 | STX7 | 1999 | YAP1 | 2039 | TRAF3IP1 | 2079 | PYGO1 |
| 1960 | RFTN1 | 2000 | MUC5B | 2040 | GPR65 | 2080 | HLA-DQB2 |

| No. | Gene symbol | No. | Gene symbol | No. | Gene symbol | No. | Gene symbol |
| --- | --- | --- | --- | --- | --- | --- | --- |
| 2081 | UBR4 | 2121 | YTHDF2 | 2161 | NR1H2 | 2201 | MUC13 |
| 2082 | CYFIP2 | 2122 | ARPC4 | 2162 | APPL2 | 2202 | TKFC |
| 2083 | ATP11A | 2123 | SPG21 | 2163 | PNMA1 | 2203 | EXOSC4 |
| 2084 | SIGLEC15 | 2124 | EVL | 2164 | ECSIT | 2204 | NCKIPSD |
| 2085 | SEMG2 | 2125 | PIAS1 | 2165 | C1QA | 2205 | RAB29 |
| 2086 | CPN2 | 2126 | CAB39 | 2166 | MUL1 | 2206 | RGCC |
| 2087 | LPXN | 2127 | AKIRIN1 | 2167 | FAM111A | 2207 | CIAO3 |
| 2088 | CYSLTR1 | 2128 | TOLLIP | 2168 | AP1M2 | 2208 | TAPBPL |
| 2089 | PTGDR2 | 2129 | KCMF1 | 2169 | DROSHA | 2209 | SLC8B1 |
| 2090 | HLA-C | 2130 | ERBIN | 2170 | AGO1 | 2210 | FBXW7 |
| 2091 | FOXL1 | 2131 | CMTM6 | 2171 | LAMTOR2 | 2211 | ACP6 |
| 2092 | FZD8 | 2132 | SLC25A38 | 2172 | PELI1 | 2212 | VAV3 |
| 2093 | CCL2 | 2133 | LAMTOR3 | 2173 | LGR4 | 2213 | ZC3H12A |
| 2094 | MAG | 2134 | DYNC1LI1 | 2174 | SNAP29 | 2214 | CHD7 |
| 2095 | PYHIN1 | 2135 | MSRB1 | 2175 | TNIP2 | 2215 | FCGRT |
| 2096 | RUNX2 | 2136 | SSBP3 | 2176 | TMEM176A | 2216 | SFTPA2 |
| 2097 | NCR2 | 2137 | CXCL14 | 2177 | KIF4A | 2217 | DUSP22 |
| 2098 | MUC7 | 2138 | DCTN4 | 2178 | PSPC1 | 2218 | PPP2R3C |
| 2099 | TRAT1 | 2139 | NUP85 | 2179 | C12orf4 | 2219 | POLR3K |
| 2100 | PRDM1 | 2140 | WRNIP1 | 2180 | SFXN1 | 2220 | TESC |
| 2101 | BTNL3 | 2141 | POLR3E | 2181 | ROGDI | 2221 | SIRT1 |
| 2102 | HLA-DMA | 2142 | RBM47 | 2182 | OAS3 | 2222 | GPATCH3 |
| 2103 | IFIT2 | 2143 | AZI2 | 2183 | SNX10 | 2223 | NCBP3 |
| 2104 | MILR1 | 2144 | PTMS | 2184 | GREM1 | 2224 | NOTCH1 |
| 2105 | FAM49B | 2145 | COMMD3 | 2185 | AGBL5 | 2225 | KLC2 |
| 2106 | ZNF675 | 2146 | COMMD9 | 2186 | EXOSC5 | 2226 | RAB17 |
| 2107 | PPIA | 2147 | PTGES2 | 2187 | ALAD | 2227 | DDX58 |
| 2108 | SEC61A1 | 2148 | NPDC1 | 2188 | EIF2B3 | 2228 | C1RL |
| 2109 | TMBIM1 | 2149 | AGPAT5 | 2189 | TBK1 | 2229 | PUS7 |
| 2110 | EIF2AK1 | 2150 | CUEDC2 | 2190 | CD320 | 2230 | DDX60 |
| 2111 | NAMPT | 2151 | DERA | 2191 | TCIM | 2231 | EDN1 |
| 2112 | TMEM30A | 2152 | HMOX2 | 2192 | NIT2 | 2232 | INAVA |
| 2113 | A2M | 2153 | CYBC1 | 2193 | MAFB | 2233 | PLAC8 |
| 2114 | RAB31 | 2154 | SMAP1 | 2194 | CDC73 | 2234 | PLEKHA1 |
| 2115 | C3 | 2155 | GSDMD | 2195 | CPPED1 | 2235 | CD248 |
| 2116 | YPEL5 | 2156 | APPL1 | 2196 | CRTC3 | 2236 | TINAGL1 |
| 2117 | GALNT2 | 2157 | CLDN1 | 2197 | DYSF | 2237 | RBP4 |
| 2118 | NPLOC4 | 2158 | IP6K2 | 2198 | RAP2C | 2238 | PODXL2 |
| 2119 | GOLPH3 | 2159 | OSTM1 | 2199 | ATP5IF1 | 2239 | SLAMF7 |
| 2120 | NOP53 | 2160 | MKNK2 | 2200 | S100A14 | 2240 | CKLF |

| No. | Gene symbol | No. | Gene symbol | No. | Gene symbol | No. | Gene symbol |
| --- | --- | --- | --- | --- | --- | --- | --- |
| 2241 | AGO4 | 2281 | COL4A3BP | 2321 | ATAD5 | 2361 | CYSLTR2 |
| 2242 | BIN2 | 2282 | TRPV1 | 2322 | CAMK1D | 2362 | TLR8 |
| 2243 | TWSG1 | 2283 | CD177 | 2323 | KLF4 | 2363 | SLC7A10 |
| 2244 | RAB8B | 2284 | AHSP | 2324 | IL17B | 2364 | DPP8 |
| 2245 | JAM2 | 2285 | DCLRE1C | 2325 | MAVS | 2365 | PGLYRP4 |
| 2246 | SPHK1 | 2286 | RTP4 | 2326 | CD244 | 2366 | IL25 |
| 2247 | SEMA4A | 2287 | SMPD3 | 2327 | DOK3 | 2367 | TSPAN14 |
| 2248 | TRIM62 | 2288 | SHPK | 2328 | IL36G | 2368 | SNX27 |
| 2249 | PDGFD | 2289 | TESPA1 | 2329 | GP6 | 2369 | TRIM8 |
| 2250 | KIF15 | 2290 | TREM2 | 2330 | BATF3 | 2370 | COLEC12 |
| 2251 | IL18BP | 2291 | PRR7 | 2331 | LRRC19 | 2371 | CTNNBL1 |
| 2252 | HERC6 | 2292 | TREML2 | 2332 | CALML5 | 2372 | TRIM34 |
| 2253 | TRPM4 | 2293 | VTCN1 | 2333 | ATP8B4 | 2373 | POLL |
| 2254 | DHX58 | 2294 | PILRA | 2334 | UBASH3A | 2374 | NOD1 |
| 2255 | KLF2 | 2295 | RAB4B | 2335 | BTNL8 | 2375 | TNFSF15 |
| 2256 | SLAMF8 | 2296 | CYP26B1 | 2336 | PLA2G2D | 2376 | IKZF3 |
| 2257 | SUSD4 | 2297 | AICDA | 2337 | CD207 | 2377 | ANGPT4 |
| 2258 | HPSE | 2298 | BARX1 | 2338 | CHST4 | 2378 | C5AR2 |
| 2259 | TRIM68 | 2299 | CLEC4E | 2339 | SLC16A8 | 2379 | KIF18A |
| 2260 | SCARA3 | 2300 | HERC5 | 2340 | HAND2 | 2380 | DCSTAMP |
| 2261 | NHEJ1 | 2301 | COLEC11 | 2341 | SIRPG | 2381 | IL21 |
| 2262 | EBI3 | 2302 | CLEC5A | 2342 | HAMP | 2382 | HOXB8 |
| 2263 | AGO3 | 2303 | ERMAP | 2343 | CLEC1B | 2383 | MZB1 |
| 2264 | TREM1 | 2304 | MMP28 | 2344 | TMEM176B | 2384 | RNASEL |
| 2265 | POLR3B | 2305 | PCID2 | 2345 | BPIFA1 | 2385 | ULBP2 |
| 2266 | APOA2 | 2306 | MCOLN1 | 2346 | TSPAN32 | 2386 | ULBP1 |
| 2267 | CLCF1 | 2307 | IL21R | 2347 | CCR10 | 2387 | CTLA4 |
| 2268 | ADA2 | 2308 | PDCD1LG2 | 2348 | RETN | 2388 | FOXP3 |
| 2269 | GCNT3 | 2309 | IL23A | 2349 | CHIA | 2389 | MPIG6B |
| 2270 | POLQ | 2310 | NOD2 | 2350 | KLRF1 | 2390 | FFAR2 |
| 2271 | DSN1 | 2311 | VPREB3 | 2351 | TNIP3 | 2391 | VPREB1 |
| 2272 | TRPV4 | 2312 | HELLS | 2352 | TBX21 | 2392 | TNFSF18 |
| 2273 | BCL11B | 2313 | C5AR1 | 2353 | DHRS7B | 2393 | IL36A |
| 2274 | ZNF16 | 2314 | CLEC2D | 2354 | IL19 | 2394 | DEFB126 |
| 2275 | KIF16B | 2315 | SLC7A9 | 2355 | ZNF580 | 2395 | PLA2G2F |
| 2276 | NCAPG2 | 2316 | TLR7 | 2356 | PLA2G3 | 2396 | BTNL2 |
| 2277 | FBXO38 | 2317 | CARD9 | 2357 | TMOD3 | 2397 | CCL24 |
| 2278 | FGG | 2318 | MUC16 | 2358 | HRH2 | 2398 | GPR32 |
| 2279 | IRAK4 | 2319 | RC3H2 | 2359 | PRG3 | 2399 | IL37 |
| 2280 | RAB20 | 2320 | ADAMTS13 | 2360 | HHLA2 | 2400 | BNIP3L |

| No. | Gene symbol | No. | Gene symbol | No. | Gene symbol | No. | Gene symbol |
| --- | --- | --- | --- | --- | --- | --- | --- |
| 2401 | TOB2 | 2461 | PRMT6 | 2501 | DPY30 | 2541 | ARL8A |
| 2402 | PLVAP | 2462 | WDR5 | 2502 | KAAG1 | 2542 | C1QC |
| 2403 | WDR61 | 2463 | RAB18 | 2503 | DLL1 | 2543 | ESAM |
| 2404 | MAGT1 | 2464 | BRI3 | 2504 | IL36B | 2544 | VSIR |
| 2405 | SLC26A6 | 2465 | LYAR | 2505 | PRELID1 | 2545 | DTX3L |
| 2406 | FCMR | 2466 | GBP3 | 2506 | DEFB103A | 2546 | NUDCD1 |
| 2407 | PYCARD | 2467 | NKAP | 2507 | GALP | 2547 | VPS26B |
| 2408 | NLRP2 | 2468 | CXCL16 | 2508 | IL1F10 | 2548 | DYNLL2 |
| 2409 | UBA52 | 2469 | EXOSC3 | 2509 | GPR174 | 2549 | DOCK8 |
| 2410 | BABAM1 | 2470 | TNFSF13B | 2510 | RNF26 | 2550 | SH3RF1 |
| 2411 | WASF2 | 2471 | ZC3H8 | 2511 | FCRL4 | 2551 | PAG1 |
| 2412 | YTHDF1 | 2472 | CARD11 | 2512 | PRXL2A | 2552 | POLR3H |
| 2413 | ARMC6 | 2473 | DLL4 | 2513 | ARHGAP9 | 2553 | STK11IP |
| 2414 | ADSS | 2474 | TNFAIP8L2 | 2514 | NFATC2 | 2554 | XIAP |
| 2415 | C6orf120 | 2475 | RNF135 | 2515 | IRF2BP2 | 2555 | UNC93B1 |
| 2416 | MARCH8 | 2476 | ITLN1 | 2516 | BRK1 | 2556 | RC3H1 |
| 2417 | TFEB | 2477 | TRIM6 | 2517 | SLC44A2 | 2557 | TMEM64 |
| 2418 | ZNF335 | 2478 | ATAD3B | 2518 | DNAJC5 | 2558 | CMTM7 |
| 2419 | MOSPD2 | 2479 | HCST | 2519 | LRRC8A | 2559 | BPIFB1 |
| 2420 | IL17RC | 2480 | CCDC88B | 2520 | SPPL3 | 2560 | ATXN1L |
| 2421 | GLRX5 | 2481 | SFTPA1 | 2521 | FAM210B | 2561 | ZNF385A |
| 2422 | GUCY1A1 | 2482 | CCL26 | 2522 | PARP14 | 2562 | TIFA |
| 2423 | SLCO4C1 | 2483 | PGM2 | 2523 | CYSTM1 | 2563 | SVIP |
| 2424 | RACGAP1 | 2484 | TLR10 | 2524 | HMGB1 | 2564 | ADSSL1 |
| 2425 | P4HTM | 2485 | GPR84 | 2525 | WDFY1 | 2565 | SPPL2A |
| 2426 | EXOC1 | 2486 | TINAG | 2526 | DPP7 | 2566 | NLRC5 |
| 2427 | IL36RN | 2487 | SLC46A2 | 2527 | ANO6 | 2567 | NRARP |
| 2428 | ACTR10 | 2488 | CD274 | 2528 | PREX1 | 2568 | ORAI1 |
| 2429 | POLM | 2489 | MOV10 | 2529 | TTC7A | 2569 | CD109 |
| 2430 | KLK5 | 2490 | TNFRSF18 | 2530 | TMEM173 | 2570 | ZFAT |
| 2431 | F11R | 2491 | BVES | 2531 | ROMO1 | 2571 | TRIM11 |
| 2432 | DBNL | 2492 | TLR9 | 2532 | CAMK2D | 2572 | MUC12 |
| 2433 | HSD3B7 | 2493 | SLC39A3 | 2533 | DAB2IP | 2573 | UNC13D |
| 2434 | APLN | 2494 | KIF2B | 2534 | SLC15A4 | 2574 | NAPRT |
| 2435 | FLVCR1 | 2495 | SUCNR1 | 2535 | ZCCHC3 | 2575 | FAM20C |
| 2436 | RAB9B | 2496 | FUT10 | 2536 | PURB | 2576 | SLFN11 |
| 2437 | ENPP3 | 2497 | CCL28 | 2537 | SIN3A | 2577 | ATP11C |
| 2438 | SURF4 | 2498 | IL20 | 2538 | EIF2AK4 | 2578 | MPEG1 |
| 2439 | RAB10 | 2499 | IFNK | 2539 | TRAPPC1 | 2579 | DOCK11 |
| 2440 | UBQLN1 | 2500 | P2RY12 | 2540 | SLC39A10 | 2580 | HVCN1 |

| No. | Gene symbol | No. | Gene symbol | No. | Gene symbol | No. | Gene symbol |
| --- | --- | --- | --- | --- | --- | --- | --- |
| 2581 | CXCL17 | 2611 | SLC7A6OS | 2641 | ZP4 | 2671 | FRMPD3 |
| 2582 | NHLRC3 | 2612 | RAB3C | 2642 | FGF10 | 2672 | CAPZA3 |
| 2583 | TRIM35 | 2613 | TMEM178A | 2643 | HOXB4 | 2673 | C17orf99 |
| 2584 | GHDC | 2614 | L3MBTL3 | 2644 | EOMES | 2674 | IL34 |
| 2585 | MUC15 | 2615 | LRRK2 | 2645 | ANKRD54 | 2675 | BPIFA2 |
| 2586 | ZBTB46 | 2616 | GBP5 | 2646 | KIF26A | 2676 | AP1S3 |
| 2587 | DTX1 | 2617 | DEFB123 | 2647 | CDH26 | 2677 | ZFPM1 |
| 2588 | RILP | 2618 | SIX4 | 2648 | MUC17 | 2678 | FCRLB |
| 2589 | IL17D | 2619 | A1BG | 2649 | COL20A1 | 2679 | SDHAF4 |
| 2590 | SUSD2 | 2620 | WIPF3 | 2650 | BPIFB2 | 2680 | CR1L |
| 2591 | EPG5 | 2621 | TMEM91 | 2651 | DEFB129 | 2681 | HES5 |
| 2592 | EEF1A1 | 2622 | MUC20 | 2652 | DEFB118 | 2682 | TIGIT |
| 2593 | MYO1G | 2623 | OSM | 2653 | DEFB127 | 2683 | PSMA8 |
| 2594 | TRIM59 | 2624 | TTBK1 | 2654 | DEFB121 | 2684 | ACOD1 |
| 2595 | TMEM179B | 2625 | SSC5D | 2655 | RNASE7 | 2685 | KIRREL3 |
| 2596 | JAML | 2626 | NFAM1 | 2656 | RAB12 | 2686 | RASGRP4 |
| 2597 | RAB37 | 2627 | RHEX | 2657 | SHLD1 | 2687 | HIST1H2BA |
| 2598 | RIPK3 | 2628 | RTKN2 | 2658 | LEO1 | 2688 | PRAM1 |
| 2599 | TICAM2 | 2629 | HMGB4 | 2659 | GBP4 | 2689 | RPS27A |
| 2600 | BLOC1S3 | 2630 | TMEM102 | 2660 | GCSAM | 2690 | PGLYRP2 |
| 2601 | LOXL3 | 2631 | LRFN5 | 2661 | NRROS | 2691 | DEFB132 |
| 2602 | TBC1D10C | 2632 | ULBP3 | 2662 | SQSTM1 | 2692 | SPACA3 |
| 2603 | UBASH3B | 2633 | PKHD1L1 | 2663 | MCEMP1 | 2693 | PYDC1 |
| 2604 | ZNF784 | 2634 | ZNF683 | 2664 | TSLP | 2694 | TMIGD2 |
| 2605 | OTULIN | 2635 | FYB2 | 2665 | VSTM1 | 2695 | ATP6V0C |
| 2606 | GAB3 | 2636 | CLEC12B | 2666 | FCRL1 |  |  |
| 2607 | BATF2 | 2637 | FCAMR | 2667 | PLPP4 |  |  |
| 2608 | IL20RB | 2638 | REG3G | 2668 | BTLA |  |  |
| 2609 | LRG1 | 2639 | GPR55 | 2669 | NLRC3 |  |  |
| 2610 | RASAL3 | 2640 | MIXL1 | 2670 | SHLD3 |  |  |

Table S4. Differentially expressed immune-related genes identified in Guangzhou cohort between pCRs and <pCRs.

| Official Symbol | Regulation in pCRs | Log2 Fold Change | *P* value |
| --- | --- | --- | --- |
| MMP1 | Upregulated | 2.2744 | 0.0066 |
| INHBA | Upregulated | 2.2112 | 0.0117 |
| SERPINE1 | Upregulated | 1.6531 | 0.0081 |
| KLK5 | Upregulated | 1.5559 | 0.0005 |
| DSG1 | Upregulated | 1.4588 | 0.0159 |
| MMP12 | Upregulated | 1.3701 | 0.0026 |
| MMP9 | Upregulated | 1.2776 | 0.0037 |
| FST | Upregulated | 1.1942 | 0.0231 |
| LGALS1 | Upregulated | 1.1901 | 0.0398 |
| AIM2 | Upregulated | 1.0682 | 0.0095 |
| PLAUR | Upregulated | 1.0271 | 0.0072 |
| CTSV | Upregulated | 1.0036 | 0.0026 |
| PTN | Downregulated | -1.0622 | 0.0474 |
| EPS8 | Downregulated | -1.0714 | 0.0095 |

Abbreviations: pCR, pathological complete response; <pCR, less than pCR.
